# Sequential use of Ad26-based vaccine regimens in NHP to induce immunity against different disease targets

**DOI:** 10.1038/s41541-022-00567-w

**Published:** 2022-11-15

**Authors:** Selina Khan, Nadine C. Salisch, Ana Izquierdo Gil, Satish Boedhoe, Karin Feddes-de Boer, Jan Serroyen, Hanneke Schuitemaker, Roland C. Zahn

**Affiliations:** Janssen Vaccines & Prevention, Leiden, The Netherlands

**Keywords:** Adaptive immunity, Cellular immunity

## Abstract

The adenovirus (Ad)26 serotype–based vector vaccine Ad26.COV2.S has been used in millions of subjects for the prevention of COVID-19, but potentially elicits persistent anti-vector immunity. We investigated if vaccine-elicited immunity to Ad26 vector–based vaccines significantly influences antigen-specific immune responses induced by a subsequent vaccination with Ad26 vector–based vaccine regimens against different disease targets in non-human primates. A homologous Ad26 vector–based vaccination regimen or heterologous regimens (Ad26/Ad35 or Ad26/Modified Vaccinia Ankara [MVA]) induced target pathogen–specific immunity in animals, but also persistent neutralizing antibodies and T-cell responses against the vectors. However, subsequent vaccination (interval, 26–57 weeks) with homologous and heterologous Ad26 vector–based vaccine regimens encoding different target pathogen immunogens did not reveal consistent differences in humoral or cellular immune responses against the target pathogen, as compared to responses in naïve animals. These results support the sequential use of Ad26 vector–based vaccine regimens targeting different diseases.

## Introduction

Adenovirus (Ad)26 vectors have been investigated in the development of vaccines for multiple infectious diseases. Adenoviruses are classified into different serotypes, with more than 70 human serotypes identified to date, and these are further divided into 7 species, A to G based on their genetic homology^1^. Two vaccines based on Ad26, which belongs to species D, are currently widely authorized: Zabdeno (in combination with a Modified Vaccinia Ankara [MVA] component, Mvabea) for the prevention of Ebola virus disease and Ad26.COV2.S for the prevention of COVID-19 disease^2–4^. Other adenovirus vectors, like ChAdOx1 and Ad5, are the basis for SARS-CoV-2 vaccines that have been developed and authorized in multiple countries for the prevention of COVID-19 as well^5,6^. Multiple additional Ad types are being explored in the development of vaccine regimens targeting human immunodeficiency virus (HIV), respiratory syncytial virus (RSV), and malaria, amongst other targets^7–11^. Pre-existing vector immunity, especially against the same Ad type, has been hypothesized to interfere with vector-mediated delivery of the vaccine-encoded antigens or to lead to immune-mediated clearance of immunogen-expressing cells. These mechanisms can potentially diminish expression of the antigen and reduce vaccine potency^12^. Pre-existing anti-vector immunity can stem from 2 sources: past exposure to the natural (or a cross-reacting) adenovirus or from a previous vaccination with the same Ad vector backbone. In either case, pre-existing immunity to the vector may negatively influence vector-based, vaccine-induced immune responses.

Decreased immune responses in the presence of pre-existing vector-targeting immune responses have been documented for some Ad vector vaccines, notably Ad5. The highly prevalent Ad5 belongs to species C and elicits high anti-vector titers in humans, making Ad5 vector–based vaccines less suitable for widespread use^13–15^. Viruses of Ad35 (type B), Ad26 (type D), and Ad48 (type D) not only have a comparatively generally lower natural seroprevalence but also lower antibody titers in seropositive individuals, although this is variable among regions. These features have made Ad26 interesting for vectorization^13^.

Published observations from clinical studies using Janssen’s Ad26-based Ebola, HIV, RSV, and COVID-19 vaccines have so far not indicated a clear negative impact of wild-type (wt) Ad26 virus–elicited, anti-Ad26 neutralizing antibody (Nab) activity in serum on Ad26 vaccine–induced immune responses after individual vaccine doses^3,7,16–21^. In the presence of vaccine-elicited anti–Ad26 vector Nabs, insert-specific immune responses are boosted by repeated administration of Ad26 vaccine encoding the same transgene^3,22^. For instance, participants in the first-in-human phase 1 study of Janssen’s COVID-19 vaccine received Ad26.COV2.S at a dose level of 5 × 10^10^ or 1 × 10^11^ virus particles (vp) in a single dose or prime-boost regimen (56-day interval between doses); Levels of Nab titers against Ad26 elicited by the first dose did not negatively impact induction of SARS-CoV-2 Nab titers on days 15 and 29 after the second dose^3^.

As both the number of vaccines based on viral vector platforms and the number of recipients of viral vector vaccines are growing, vaccination-elicited anti-vector immunity will likely increase. In the future, over their lifetime, individuals could conceivably receive multiple viral vector–based vaccines with the same vector backbone encoding different antigens. Therefore, it is important to understand the impact of vaccine-elicited anti-vector immunity on the insert-specific immune response and ultimately the efficacy of subsequently administered vaccines using the same vector. Here, we determined whether pre-exposure to Ad26 vector–based vaccines critically influenced the immune responses induced by subsequent vaccination with Ad26 vectors encoding different pathogen immunogens in non-human primates (NHPs).

## Results

**Fig. 1:**
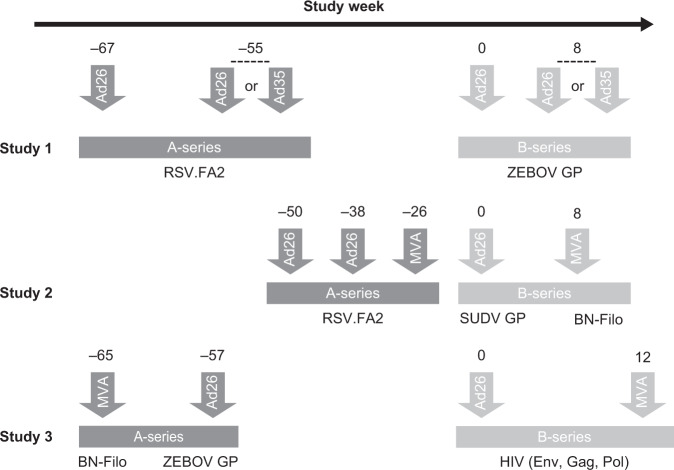
Summary of study designs and immunization regimens. Generic study NHP designs: Ad26 vaccines administered during A-series as a homologous or heterologous regimen with Ad35 or MVA was followed 26 to 57 weeks later by B-series of the same vector-backbone regimen. In all studies, the vaccines given during A- and B-series administration encoded different antigens. Study 1: Animals pre-exposed to Ad26 or Ad35 encoding a RSV.FA2 antigen were dosed 55 weeks after the last vaccination (A-series) with the same sequence of Ad26- or Ad35-encoding EBOV GP in B-series (dose 1, week 0; dose 2, week 8). Control animals received the same EBOV GP–encoding homologous or heterologous regimens at week 0 and week 8 in B-series. Study 2: Animals pre-exposed to Ad26 and MVA encoding a RSV.FA2 antigen were dosed 26 weeks after the last vaccination (A-series) with the same sequence of Ad26 or MVA, with Ad26-encoding SUDV GP (dose 1, week 0) and MVA-BN-Filo (dose 2, week 8), respectively, during the B-series. Control animals received the same SUDV GP–encoding vaccines at week 0 and week 8 in B-series. Study 3: Animals pre-exposed to MVA-BN-Filo and Ad26-encoding EBOV GP were dosed 57 weeks after the last vaccination (A-series) with the same sequence of Ad26 or MVA encoding a mosaic of HIV Env, Gag, and Pol antigens (dose 1, week 0; dose 2, week 12) in B-series. Control animals received the same vaccine (Ad26 then MVA) at week 0 and week 12 in B-series. Details of the study designs are provided in Supplementary Fig. 1.

We conducted 3 independent studies in cynomolgus macaques. Ad26 vector–based vaccines were administered as a 2- or 3-dose homologous regimen or as part of a heterologous regimen in combination with Ad35- or MVA vector–based vaccines (Fig. 1, Supplementary Fig. 1). Animals first received a homologous or heterologous vaccine regimen (referred to as “A-series”), then 26 to 57 weeks later received the same vector regimen but with vectors encoding different transgenes (“B-series”) to mimic a clinical situation where individuals would receive multiple different Ad26 vector–based vaccine regimens during their lifetime. Animals dosed in the A-series are referred to as “pre-exposed” in this manuscript, whereas control animals only dosed in the B-series are referred to as “unexposed.” The annotation “[rep]” after the vaccine regimen refers to “repeated dosing” (e.g., animals dosed in both the A-series and B-series). Peripheral blood mononuclear cells (PBMCs) and serum were collected from animals at defined time points during the A-series and B-series to longitudinally assess the cellular and humoral immune responses directed towards the vector backbone and the antigen.

### Ad26 vaccine–elicited anti-vector Nab and T-cell responses

**Fig. 2:**
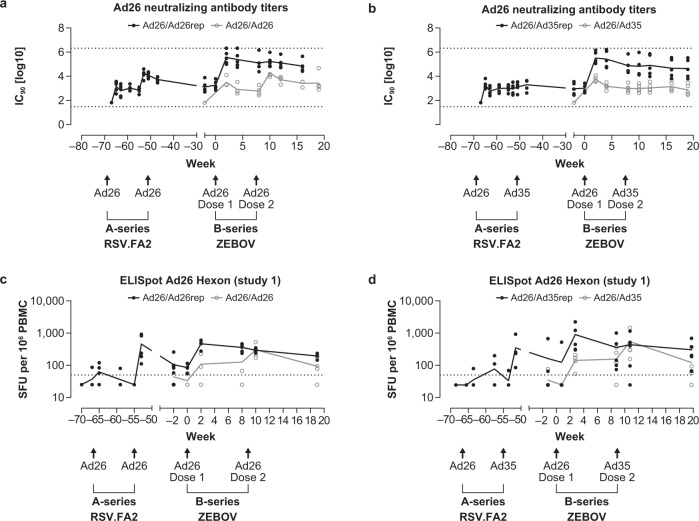
Anti-Ad26 vector responses in animals dosed with Ad26 vectors. Ad26 (**a**, **b**) Nab titers were determined using an Ad26-based VNA with sera obtained from animals in the indicated groups from study 1. Each symbol represents 1 animal (filled circle, animals dosed in A-series with Ad26; open grey circle, animals not dosed in A-series). Dotted lines depict ULoD (log10 highest serum dilution in the assay, 1/65536 for Ad26) and LLoD (log10 lowest serum dilution in the assay, 1/64 for Ad26), respectively. *N* = 4 to 5 animals per group (**c**, **d**). Ad26 hexon-specific T-cell responses were measured by IFNγ ELISpot using PBMCs stimulated with peptide pools covering Ad26 hexon sequences. The dotted line corresponds to a threshold of 50 SFU/106 PBMCs. The horizontal lines represent the mean group response. Animals that received an Ad26 and/or Ad35 vaccine during the A-series are annotated as Ad26/Ad26rep or Ad26/Ad35rep.

First, we determined the level of anti-Ad26 Nabs in the serum of animals after the A-series of the 2-dose homologous Ad26/Ad26 or heterologous Ad26/Ad35 vaccine vector regimen. Ad26 Nabs were elicited in all animals after the first dose, increased after a second dose with Ad26 but not Ad35, and diminished or remained detectable at lower levels compared to the peak response for >50 weeks after the last Ad26 vaccine dose of the A-series (Fig. 2a). Administration of the first, but not the second, dose of the Ad26 vaccine encoding EBOV GP of the B-series further amplified Ad26 Nab titers in pre-exposed animals. Ad26 Nab titers elicited in the B-series by the 2 doses of Ad26.ZEBOV in unexposed animals were in the same range as those induced during the A-series of Ad26 RSV-FA2.

Heterologous vaccination with the Ad26/Ad35 or Ad26/MVA regimens elicited comparable levels of anti-Ad26 Nab titers to those elicited by the homologous Ad26/Ad26 vaccine regimen after the first vaccination dose; as expected, higher anti-Ad26 Nab titers were seen with the Ad26/Ad26 vaccine regimen after the second dose of Ad26 compared with after the second dose of Ad35 or MVA in the A-series (Fig. 2b, Supplementary Fig. 2).

T-cell responses targeting the most abundant adenoviral structural protein, hexon, elicited by the Ad26 and Ad35 vectors were assessed in study 1 by an interferon (IFN)γ enzyme-linked immunosorbent spot assay (ELISpot). Five peptide pools containing shared and specific peptides for Ad26 and Ad35 hexon protein were used. Responses are reported as either Ad26 hexon–specific (sum of 2 Ad26-specific peptide pools) or pan-Adeno Hexon response (sum of the responses to the 5 peptide pools).

Ad26 hexon protein–specific T-cell responses were elicited by Ad26 vaccination (Fig. 2c, d) and reached peak IFNγ ELISpot counts of an average of 456 spot forming units (SFU)/10^6^ cells 2 weeks after the last Ad26 dose in the A-series, followed by a considerable contraction to 85 SFU/10^6^ cells by the time of the B-series 50 weeks later. In line with Ad26 Nab titers, first dosing with Ad26.ZEBOV in the B-series resulted in a sharp increase in Ad26 hexon–specific IFNγ ELISpot responses in pre-exposed animals (Ad26/Ad26rep: 466 SFU/10^6^ cells; Ad26/Ad35rep: 918 SFU/10^6^ cells), while a lower response was seen in previously unexposed animals (Ad26/Ad26: 109 SFU/10^6^ cells; Ad26/Ad35: 144 SFU/10^6^ cells). The second dose of Ad26 or Ad35 in the B-series 8 weeks later did not further increase Ad26 hexon–specific T-cell responses in pre-exposed animals (Ad26/Ad26rep: 293 SFU/10^6^ cells; Ad26/Ad35rep: 445 SFU/10^6^), whereas a small increase was observed in the unexposed animals (Ad26/Ad26: 303 SFU/10^6^; Ad26/Ad35: 558 SFU/10^6^) at week 10, similar to the A-series response. A similar trend was observed for pan-Adeno Hexon response (Supplementary Fig. 2).

### Ad26 vector pre-exposure has no consistent impact on antigen-specific responses induced by 1 dose of Ad26

Having established that neutralizing Ad26 antibodies and hexon-specific T cells were present after the vaccination A-series, we next investigated the antigen-specific immune response following the B-series of Ad26 vaccines in a homologous regimen or in a heterologous regimen with Ad35 or MVA vaccine vectors.

**Fig. 3:**
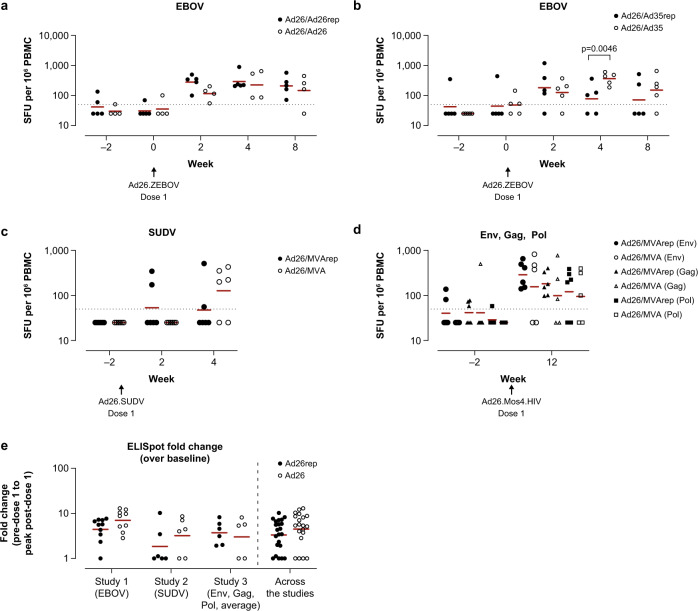
Ad26 vaccine–elicited vector-immunity has minor impact on cellular immune responses induced by a first dose of the B-series Ad26 vaccines in cynomolgus macaques. IFNγ ELISpot responses in PBMC (**a**–**d**) were determined after stimulation with pools of 15-mer peptides overlapping by 11 amino acids, covering the protein sequence of EBOV GP (EBOV) (**a**, **b**); SUDV GP (SUDV) (**c**); and Env, Gag, and Pol (**d**). Shown are background-subtracted values per animal of animals receiving Ad26 regimen encoding EBOV GP (**a**, **b**), regimen encoding SUDV GP (**c**), and regimen encoding Mos4.HIV (**d**). Animals that received an Ad26 vaccine during the A-series are annotated as Ad26/Ad26rep (**a**), Ad26/Ad35rep (**b**), and Ad26/MVArep (**c**, **d**). In **a**–**d**, the dotted line represents the assay threshold of 50 SFU/10^6^ PBMC. Red bars depict geometric group means. In (**e**), the fold-change in IFNγ SFU/10^6^ PBMCs is depicted and corresponds to the change in response comparing pre–dose 1 to peak response post–dose 1 per animal. Ad26rep refers to animals vaccinated in the A-series, and Ad26 to animals that were not vaccinated during the A-series. The red horizonal line is the geometric mean. For all 3 studies, the baseline (pre–dose 1) was defined as the time point just prior to dosing (studies 1–3, week –2). Pairwise comparison of the difference between pre-exposed animals and unexposed animals per time point was performed for data in (**a**–**d**), summarized in Supplementary Table 1. An ANOVA was performed over the fold-changes per study and across the 3 studies over the data shown in (**e**), summarized in Supplementary Table 3.

Across all 3 studies, no major impact of pre-existing vaccine-elicited Ad26 immunity on absolute antigen-specific cellular immunity, as measured by IFNγ ELISpot, was detected after the first Ad26 dose in the B-series (Fig. 3a, c, e). This observation held true irrespective of whether the immune response was directed against a membrane-bound vaccine antigen such as the glycoproteins of EBOV or SUDV, the HIV envelope protein (Env; Fig. 3a–d), or intracellular antigens such as HIV group–specific antigen (Gag) or HIV polymerase protein (Pol). The only statistically significant difference was observed in the EBOV response at week 4, where a higher IFNγ ELISpot response was seen in Ad26/Ad35-dosed compared to Ad26/Ad35rep-dosed animals (*p* = 0.0046; Tobit model; Fig. 3b, Supplementary Table 1). However, no significant differences were detected in the relative fold-change in antigen-specific IFNγ ELISpot responses at the post–dose 1 peak response over baseline response for any of the antigens between Ad26 pre-exposed and unexposed animals (study 1 comparison: Ad26rep vs unexposed, *p* = 1.0000; study 2: Ad26rep vs unexposed, *p* = 0.3400; study 3: Ad26rep vs unexposed, *p* = 0.6700, analysis of variance [ANOVA]; Fig. 3e, Supplementary Table 3).

**Fig. 4:**
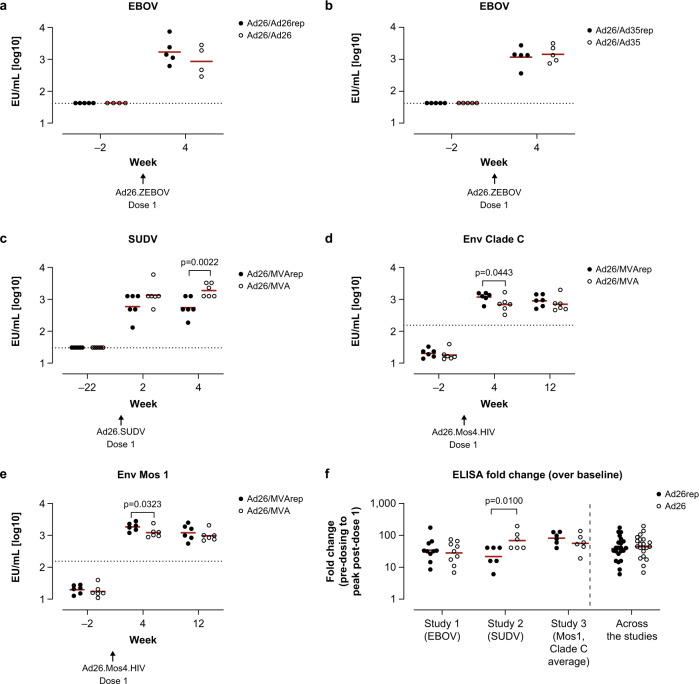
Ad26 vaccine–elicited vector-immunity has a minor impact on humoral immune responses induced by a first dose of the B-series in cynomolgus macaques. Binding serum antibody concentrations were measured by ELISA for each regimen specific for EBOV glycoprotein (GP) (**a**, **b**), SUDV GP (**c**), HIV Env Clade C (**d**), and HIV Env Mos1 (**e**). Shown are geometric group means of the log10 EU/mL values determined relative to an assay standard. Dotted lines represent the LLoD/LLoQ. For (**a**, **b**), the LLoQ of the human assay of 1.62log10 was used. For (**c**), the LLoD was defined as 1.48log10 (EU/mL), which was measured for the pre-dosing samples in the SUDV GP–specific assay^36^. For (**d**, **e**), the LLoQ of the human assay of 2.19log10 was used, whereas the ULoQ of the human assay was 3.69log10^31^. The fold-change in antibody concentrations is depicted in (**f**) and corresponds to the change in response comparing pre–dose 1 to peak response post–dose 1 per animal. “Ad26rep” refers to animals vaccinated in the A-series, and “Ad26” refers to animals that were not vaccinated during the A-series. Pairwise comparison of the difference between pre-exposed animals and unexposed animals per time point was performed for data in (**a**–**e**), summarized in Supplementary Table 2. An ANOVA was performed over the fold-changes per study and across the 3 studies over the data shown in (**f**), summarized in Supplementary Table 3.

As observed for cellular responses, pre-exposure did not result in a consistent decrease of absolute antigen-specific humoral immune responses induced by the first Ad26 dose in the B-series, as measured by immunoglobulin G (IgG) enzyme-linked immunosorbent assay (ELISA), although the impact was more nuanced (Fig. 4, Supplementary Table 2). In study 1, higher group mean EBOV-binding antibody concentration was seen in the Ad26/Ad26rep group compared to the Ad26/Ad26 group (Fig. 4a), although it did not reach statistical significance (Supplementary Table 2). Similarly, no impact of pre-existing immunity on EBOV-binding antibodies was observed in animals who received Ad26/Ad35 in the A-series (Fig. 4b). There was a transient, but inconsistent, difference observed in the absolute EBOV Nab titers in pre-exposed animals (groups Ad26/Ad26rep and Ad26/Ad35rep) compared with unexposed animals (groups Ad26/Ad26 and Ad26/Ad35; Supplementary Fig. 3a–b). There was a higher response in Ad26/Ad26-dosed compared to Ad26/Ad26rep-dosed animals that reached statistical significance at week 10 and week 12 (*p* = 0.0188 and *p* = 0.0138, respectively; Tobit model), but not at week 4 and week 19 (Supplementary Table 2).

The impact of pre-existing Ad26 immunity on absolute SUDV GP–binding antibody concentration (study 2) was transient, with a statistically significant lower response observed at week 4 (*p* = 0.0022; Tobit model), but not at week 2 (*p* = 0.0977; Fig. 4c, Supplementary Table 2). By contrast, binding antibody concentrations to Env Clade C and Env Mos1 (study 2) were transiently significantly higher in animals with pre-existing immunity at week 4 (Clade C: *p* = 0.0443; Mos1: *p* = 0.0323; Tobit model), but not at week 12 (Clade C: *p* = 0.3629; Mos1: *p* = 0.3895; Fig. 4d–e). Analysis of the relative fold-change in antibody concentrations showed that for studies 1 and 3 there was no statistically significant difference between animals with or without pre-existing Ad26 immunity (study 1 comparison: Ad26/Ad26rep vs Ad26/Ad26, p = 0.5371; study 3: Ad26/MVArep vs Ad26MVA, *p* = 0.2891; ANOVA); for study 2, a significantly lower fold-increase was observed in animals with pre-existing Ad26 vaccine–induced immunity (Ad26/Ad26rep vs Ad26/Ad26, *p* = 0.0189; ANOVA; Fig. 4f, Supplementary Table 3). For study 1, next to binding antibodies, EBOV neutralizing titers were also evaluated and tended to be lower in animals with pre-existing Ad26 immunity (Supplementary Fig. 3), with significant differences seen compared with the non-exposed animals at week 10 (Ad26/Ad26rep vs Ad26/Ad26, *p* = 0.0188; ANOVA) and week 12 (Ad26/Ad26rep vs Ad26/Ad26, *p* = 0.0138; ANOVA), but not at week 4 and week 19, and also did not reach statistical significance when comparing the fold-change from pre-dosing to post–dose 1 (Supplementary Fig. 3).

Together, these data suggest pre-existing vaccine-elicited Ad26 immunity was associated with temporary and inconsistent effects on the cellular and humoral antigen-specific responses induced by the first Ad26 in B-series, which did not amount to a consistent or substantial negative impact.

### Vector pre-exposure has no consistent impact on vaccine antigen–specific responses induced by a second dose of vaccine, irrespective of vector

**Fig. 5:**
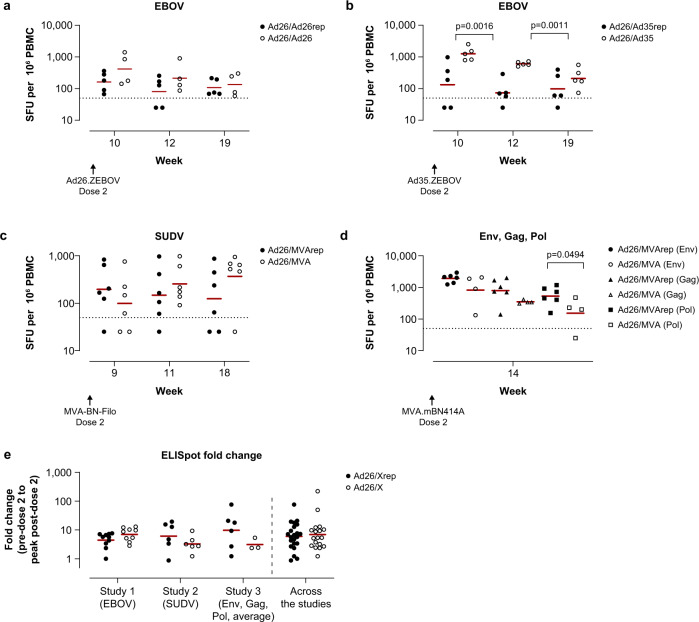
Heterologous vectors boost Ad26 vaccine–induced cellular immune responses in the presence of pre-existing Ad26 immunity in cynomolgus macaques. IFNγ ELISpot responses in PBMC were determined as described in the Fig. 3 legend, with peptide pools for EBOV GP (**a**, **b**); SUDV GP (**c**); and HIV Env, Gag, and Pol (**d**). Shown are background-subtracted values per animal of animals receiving an Ad26/Ad26 (**a**) or Ad26/Ad35 (**b**) regimen encoding EBOV GP; Ad26 encoding SUDV GP and MVA encoding SUDV GP, EBOV GP, MARV GP (**c**); or Ad26/MVA encoding Env, Gag, and Pol of HIV (**d**). Animals that had received an Ad26 vaccine during the A-series are annotated as Ad26/Ad26rep (**a**), Ad26/Ad35rep (**b**), or Ad26/MVArep (**c**, **d**). In (**a**–**d**), the dotted line represents the assay threshold of 50 SFU/10^6^ PBMC. In (**e**), the fold-change is depicted and corresponds to the change in responses comparing pre–dose 2 to peak response post–dose 2 per animal. Ad26/Xrep refers to animals vaccinated in the A-series, and Ad26/X refers to animals that were not vaccinated during the A-series. The red horizonal line is the geometric mean. Pairwise comparison of the difference between pre-exposed animals and unexposed animals per time point was performed for data in (**a–d**), summarized in Supplementary Table 1. An ANOVA was performed over the fold-changes per study and across the 3 studies over the data shown in (**e**), summarized in Supplementary Table 3.

Next, we assessed the immune response induced after the second vaccine dose in the B-series, consisting of vaccines based on either Ad26, Ad35 (study 1), or MVA (studies 2 and 3). In study 1, higher absolute EBOV-specific IFNγ ELISpot responses were induced in unexposed compared to pre-exposed animals receiving the heterologous Ad26/Ad35 regimen, which was most pronounced at week 10 and week 12 (week 10: *p* = 0.0016; week 12: *p* = 0.0011; Tobit model; Supplementary Table 1), but not for the homologous Ad26/Ad26 regimen (Fig. 5a, b). There was a comparable fold-increase in EBOV GP–specific IFNγ ELISpot responses above pre-boosting levels following a second (homologous) dose of Ad26.ZEBOV at week 8 in animals with and without pre-existing Ad26 immunity (Fig. 5e, Supplementary Table 3).

For the heterologous Ad26/MVA schedule (study 2), the magnitude of SUDV-specific IFNγ-producing cells after MVA-BN-Filo dosage at week 9 was comparable for animals pre-exposed to Ad26 and unexposed, and thereafter a trend for an increase of SUDV-specific IFNγ-producing cells over time in the Ad26-unexposed group was observed. A similar increase was not observed in the animals with pre-existing immunity to Ad26 and MVA, although the difference between the pre-exposed and unexposed animals did not reach statistical significance (Fig. 5c, Supplementary Table 2). Furthermore, there was no clear difference in the relative fold-change of the peak response between pre-exposed and unexposed animals (*p* = 0.2793; ANOVA; Fig. 5e, Supplementary Table 3).

In study 3, in which animals were dosed with Ad26.Mos4.HIV/MVA-mBN414A (Ad26/MVA rep group), higher levels of the individual, absolute Env-, Gag-, and Pol-specific IFNγ ELISpot responses were induced in pre-exposed versus unexposed animals after the second dose, which reached statistical significance for the Pol-specific response (*p* = 0.0494; Tobit model), but not for the Env- or Gag-specific responses (Fig. 5d, Supplementary Table 1). On the other hand, a significant difference was detected between pre-exposed and unexposed animals in terms of the relative fold-increase in individual Env-specific responses (*p* = 0.0107; ANOVA; Supplementary Table 3), but not in the individual Gag antigen (*p* = 0.7088; ANOVA), individual Pol antigen (*p* = 0.0788; ANOVA), or average fold-change of the 3 antigens (*p* = 0.0877; ANOVA; Fig. 5e, Supplementary Table 3).

A pooled analysis of all 3 studies mirrored the results from the individual studies, showing no statistically significant difference in the fold-change of the cellular immune response in animals pre-exposed to Ad26, Ad35, or MVA vectors compared to unexposed animals (*p* = 0.4530; ANOVA).

**Fig. 6:**
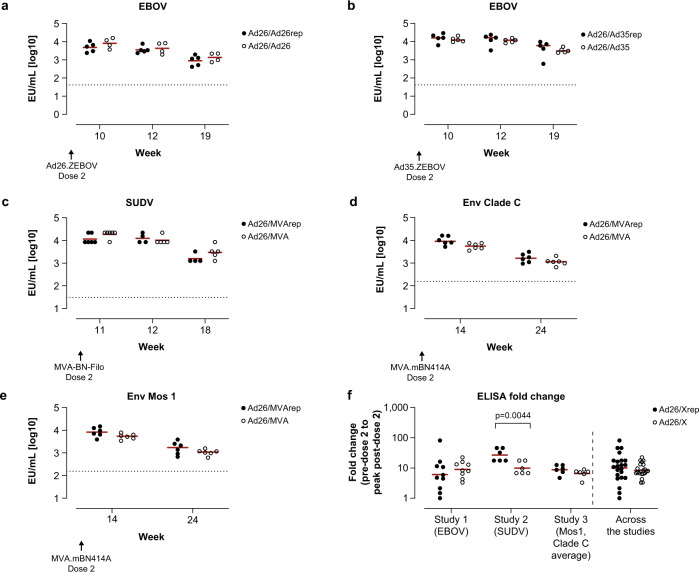
Heterologous vectors boost Ad26 vaccine–induced humoral immune responses in the presence of pre-existing Ad26 immunity in cynomolgus macaques. Binding serum antibody concentrations were measured by ELISA for each regimen specific for EBOV GP (**a**, **b**), SUDV GP (**c**), HIV Clade C Env (**d**), and HIV Mos1 Env (**e**), as described in the Fig. 4 legend. Shown are geometric group means of the log10 EU/mL values determined relative to an assay standard. Dotted lines represent the LLoD/LLoQ. For (**a**, **b**), the LLoQ of the human assay of 1.62log10 was used. For (**c**), the LLoD was defined as 1.48log10 (EU/mL), measured for the pre-dosing samples in the SUDV GP–specific assay^36^. For (**d**, **e**), the LLoQ of the human assay of 2.19log10 was used, whereas the ULoQ of the human assay was 3.69log10^31^. The fold-change in antibody concentrations is depictured in (**f**) and corresponds to the change in response comparing pre–dose 2 to peak response post–dose 2 per animal. Ad26/Xrep refers to animals vaccinated in the A-series, and Ad26/X refers to animals that were not vaccinated during the A-series. The red horizonal line is the geometric mean. Pairwise comparison of the difference between pre-exposed animals and unexposed animals per time point was performed for data in (**a**–**e**), summarized in Supplementary Table 2. An ANOVA was performed over the fold-changes per study and across the 3 studies over the data shown in (**d**), summarized in Supplementary Table 3.

For vaccine-induced humoral immune responses, specific antibody concentrations induced by the final vaccine dose in the different regimens were similar in animals with and without pre-existing Ad26, Ad35, and MVA immunity at all observation time points (Fig. 6a–e).

The relative fold-change from the pre–dose 2 response to the post–dose 2 peak response showed a significantly higher anti-SUDV–specific antibody response in animals pre-exposed to Ad26 and MVA than in unexposed animals (*p* = 0.0044; ANOVA). For the other vaccination schedules, no significant difference in fold-change between pre-exposed and unexposed animals was observed for the humoral immune response (Fig. 6f). Similarly, pooled results across the 3 studies (Fig. 6f) showed no statistically significant difference when comparing the relative fold-change induced in pre-exposed versus unexposed animals (Ad26/Xrep vs Ad26/X, where X refers to either Ad26, Ad35, or MVA; *p* = 0.5046; ANOVA).

Absolute EBOV GP Nab titers in animals pre-exposed and unexposed to Ad26 and/or Ad35 in study 1 were comparable, with no statistically significant differences observed in the relative fold-change from pre–dose 2 to peak response post–dose 2 (Ad26/Ad26rep vs Ad26/Ad26: p = 0.2780; Ad26/Ad35rep vs Ad26/Ad35: *p* = 0.1876; ANOVA; Supplementary Fig. 3). Importantly, RSV binding antibodies and neutralizing antibodies induced during the A-series dosing in study 1 and 2 were not reduced after the B-series dosing^9^ and Supplementary Fig. 4, respectively.

## Discussion

Adenoviruses have been vectorized and widely investigated for use in prophylactic and therapeutic vaccines that target a wide range of pathogens and for other indications like gene therapy^23^. We have selected Ad26 for the development of vector-based vaccines due to its relatively low seroprevalence and low neutralization titers in seropositive individuals. So far, we have not observed an impact of natural pre-existing Ad26 Nabs on the potency of Ad26-based vaccines^3,7,16–18,20,21^. However, a remaining important question is whether vaccine-elicited vector immunity may impact the potency of subsequently used Ad26-based vaccines that target different pathogens. This is particularly important in the fight against the COVID-19 pandemic where a considerable part of the global population received SARS-CoV-2 vaccines based on Ad26.

NHPs are considered an excellent immunogenicity model for Ad26-based human vaccines, as demonstrated with the mosaic Ad26/Ad26 + gp140 HIV-1 and the Ad26-based Ebola vaccines, which induced comparable and robust immune responses in humans and rhesus or cynomolgus macaques, respectively^24^. In fact, the Ad26-based Ebola vaccine regimen was licensed based on comparing immunogenicity between cynomolgus macaques and humans to derive an estimate for the protective effect of the vaccine based on protection against Ebola virus disease in the animal model^25^. While wt Ad26 does not naturally infect macaques^13^, and hence no natural Ad26-specific immunity is present in macaques, anti-Ad26 vector–elicited Nab titers post-vaccination reached at least the same magnitude as in humans^18^, as shown in this manuscript and previous studies^9,13^, making it a suitable species for a deeper understanding of anti-vector immunity in the context of repeated Ad26 vaccination.

Three studies in NHPs were conducted to determine whether vaccine-elicited immunity to Ad26 had an impact on the immune responses to subsequent Ad26-based vaccines encoding different immunogens. Despite relatively high levels of humoral and cellular anti-vector responses present prior to the dosing of the second Ad26 vaccination series, no clear or consistent impact of pre-existing vector-elicited Ad26 immunity on humoral or cellular immune responses against the target immunogen was observed. Neither immunity induced by the first Ad26 dose alone nor by the complete second regimen was consistently affected. Importantly, a first dose of Ad26 vaccine induced a vaccine antigen–specific immune response of similar magnitude in animals pre-exposed to homologous Ad26/Ad26 or heterologous Ad26/Ad35 or Ad26/MVA regimens, as compared to naïve animals, suggesting there are no additive effects of pre-existing anti-vector immunity elicited by repeated previous Ad26 exposures for the dose range tested.

While pre-existing anti-vector immunity is a potential concern for reuse of viral vector–based vaccines in general, not all appear to be equally impacted^26^. Vaccines based on MVA, for example, were shown to be affected by pre-existing immunity to orthopoxviruses induced by previous vaccination with a smallpox vaccine^27^. However, those vaccinia-specific binding and Nabs appear to persist predominantly in those vaccinated during childhood as compared to those vaccinated during adulthood^28^. Confirming these observations in humans, immune responses induced by a second MVA-containing vaccine regimen in adult NHPs were not negatively impacted in our studies, but augmented by a second dose.

Our results on the effect of pre-existing anti-vector immunity contrast, however, with previous observations in NHPs in which Ad5 vaccine–elicited pre-existing immunity strongly inhibited vaccine-specific antibody responses^29^. Similarly, the marked impact of pre-existing natural Ad5 immunity in humans due to previous Ad5 viral infection has been well documented and was consistently observed across a range of antigens and applications^14,30–34^. In contrast, the limited human data currently available from individual clinical trials with Ad26 vector–based vaccines do not suggest a similar clear and consistent impact of pre-existing immunity to Ad26 arising from natural Ad26 viral exposure^3,16,35–37^. Similarly, repeated vaccination with Ad26 vector–based vaccines in homologous vaccination regimens have so far resulted in consistent boost responses to a second or further subsequent dose of the same Ad26 vector regimen in clinical studies^3,16,19,20,24,38^. Emary and colleagues^39^ have shown that comparable levels of anti-spike IgG antibodies were elicited by the ChAdOx1 nCoV-19 vaccine in participants of a phase 2/3 study with and without prior ChAdOx1 (encoding a different antigen) vaccine exposure. This indicates pre-existing anti-vector responses did not hamper insert-specific immune responses for this non-human group E Ad vector, although the number of study participants was relatively small (*n* = 10 for pre-exposed; *n* = 48 for unexposed individuals)^39^.

The demonstrated difference between patterns of immune response induced by different Ad serotypes, such as Ad5, CHAdOx1, and Ad26, in pre-exposed individuals remains unexplained to date. It can be speculated that the variable sensitivity to pre-existing immunity likely reflects the highly disparate biologic mechanisms of cell entry, receptor binding, trafficking, endosomal functioning, and cell- or receptor-tropism used by the different vectors^26^. Ad26, along with Ad48, Ad35^40^, and Ad28^41^ vectors, has been reported to induce a distinct innate immune profile with high expression of type I IFN genes; for Ad35 and Ad28, the IFNα expression was shown to negatively correlate with antigen expression^41^. In contrast, C-type vectors like Ad5 and ChAd3 induce lower expression of type I IFN genes and a higher antigen expression^40,41^. How strongly these different characteristics are mechanistically associated with an adenoviral vector’s sensitivity to pre-existing immunity is not known. However, it is striking that a strong and persistent insert-specific adaptive immune response is induced by C-type vectors in naïve animals^42^, while subsequent dosing with Ad5 vectors abolishes the antigen-specific response in animals with pre-existing Ad5 immunity^29,43^. Interestingly, Ad5 seroprevalence by natural infection is much higher, along with higher Ad5 neutralizing titers in seropositive individuals; this is in contrast to Ad26, which has lower seroprevalence and lower Ad26 neutralizing titers in seropositive individuals^13,44^. Further research will be needed to understand how pre-existing immunity influences the different Ad serotypes and which factors modulate its impact on vaccine potency.

Our investigation has multiple limitations. One limitation is the relatively small sample size of the individual studies, which could mask overall trends due to differences in the individual study designs and vector antigens encoded by the different vaccines. To address this shortcoming, we combined the 3 independent studies to statistically investigate general patterns across multiple vaccine antigens, which indicated no overall trend for a consistent negative impact of vaccine elicited anti-Ad26 immunity. This analysis does not address the question of how far pre-existing anti-vector immunity affects the efficacy of subsequently administered vaccines. However, levels of adaptive immunity can be utilized to predict protection, as we have previously shown that for Ad26-based Ebola and HIV vaccines, binding antibodies are a strong predictor of protection^17,25^ and in addition HIV-specific IFNγ cellular immune responses correlated with efficacy in Ad26 HIV–immunized NHPs^17^. Given the substantial use of the Ad COVID-19 vaccines ChAdOx1-SARS-COV-2 (Vaxzevria) and Ad26.COV2.S (Janssen COVID-19 Vaccine), evidence of potential interference could be collected in the context of clinical efficacy studies of other Ad26-based vaccines.

In conclusion, we have shown here that while Ad26 vector–based vaccines elicit transient high levels of anti-Ad26 Nab– and hexon-specific T cells that persisted at lower levels, these did not have a substantial nor consistent impact on antigen-specific cellular and humoral immunity induced by a second administration of Ad26 vector–based vaccine regimens against a different pathogen in NHPs. These results support investigation of the sequential use of Ad26 vector–based vaccine regimens in humans.

## Methods

### Adenoviral vectors

Replication-incompetent, E1/E3-deleted, recombinant Ad26 and Ad35 vectors were engineered using the AdVac^®^ system (Janssen)^11,45^, as described in detail for Ad26.ZEBOV, Ad35.ZEBOV, Ad26.SUDV, and Ad26.Mos4.HIV^7,24,46^. Ad26.Mos4.HIV consists of 4 Ad26 vectors: Ad26.Mos.1.Env (encodes a mosaic insert of the Env protein sequence), Ad26.Mos2S.Env (encodes modified Mos2 HIV-1 Env protein sequence), Ad26.Mos1.Gag-Pol (encodes Mos1, HIV-1 Gag and Pol protein), and Ad26.Mos2.Gag-Pol (encodes Mos2 HIV-1 Gag and Pol protein). Briefly, codon-optimized genes encoding the relevant transgene were inserted into the E1 position of the Ad genomes under transcriptional control of the human cytomegalovirus promoter and the SV-40 polyadenylation sequence.

Cloning, rescuing, and manufacturing of the replication-deficient Ad vectors using the complementing cell line PER.C6^®^ (Janssen)^45^. Viral particles (vp) titers in the viral preparations were quantified by measurement of optical density at 260 nm^47^, and infectivity (expressed as infectious units [IU]) was assessed by tissue culture infective dose 50%^48,49^. The vp/IU ratio was between 1:1 and 6:1 for the viral preparations. Ad-mediated expression of the various transgenes was confirmed by Western blot analysis of cell culture lysates from infected A549 cells or by polymerase chain reaction.

### MVA vectors

MVA-BN-Filo is a trivalent recombinant MVA strain Bavarian Nordic (MVA-BN)–based filovirus vaccine directed against Marburg virus and Ebola virus infection. The full-length coding sequences for GP antigens of MARV Musoke, EBOV Mayinga, and SUDV Gulu, as well as the nucleoprotein from Taï Forest Ebola virus, were codon optimized, synthesized (GeneArt, Regensburg, Germany), inserted into MVA-BN^46^. MVA-mBN414A compromising a single MVA-BN vector was genetically engineered to encode Mos1.Env, Mos2S.Env, Mos1.Gag-Pol, and Mos2.Gag-Pol HIV-1 protein sequences^17^. MVA-RSV.FA2 (MVA-mBN235A) is a monovalent vaccine comprising a single MVA-BN vector genetically engineered to encode the F fusion protein of RSV strain A2 under the synthetic early-late promoter PrS. Primary chicken embryo fibroblast cells used for recombinant live, attenuated MVA viral vector–based vaccine generation and production were prepared from embryonated eggs and maintained in serum-free conditions.

### Animals and housing

For study 1, a total of nineteen 5- to 6-year-old, healthy female cynomolgus macaques (*Macaca fascicularis*) of Vietnamese origin (body weight 3–8 kg at study start) were rolled over from a previous study^9^. The animals were originally purchased from Covance (Alice, TX, USA). Five of the animals had received previous vaccination with an Ad26/Ad26 homologous regimen, and 5 had previously received an Ad26/Ad35 heterologous regimen; the insert encoded by these vectors was RSV.FA2^9^. Nine other animals were vaccine-naïve upon enrollment.

For study 2, twelve 4- to 5-year-old, healthy female cynomolgus macaques of Mauritian origin (body weight 3–7 kg at study start) were rolled over from a previous study and purchased by Charles River Edinburg. Six of the animals had previously received 2 doses of an Ad26 vector expressing a fusion protein of RSV.FA2 and Gaussian luciferase and a dose of MVA encoding RSV.F_A2_, and 6 were vaccine-naïve upon enrollment.

For study 3, twelve 4- to 7-year-old, healthy male and female cynomolgus macaques of Chinese origin (body weight 3–7 kg at study start) were rolled over from a previous study and purchased by Charles River Reno NV and Alpha Genesis Inc. Six of the animals had previously received an MVA-Ad26 heterologous regimen; the inserts of those vectors were EBOV GP for Ad26 and MVA-BN-Filo. Six other animals were vaccine-naïve upon enrollment.

All animals were kept in a biosafety level 2 facility under specific pathogen-free conditions after screening negative for *Mycobacterium tuberculosis*, simian immunodeficiency virus, simian retrovirus, and simian T-lymphotropic virus. Screening included herpes B virus and measles serology.

Animals in study 1 and study 3 were pair-housed in groups of 2 or 3 animals in stainless steel cages placed in study-dedicated, USDA- and OLAW-approved rooms, while animals in study 2 were socially housed in groups of 6 in 2-story gang pens, which were also OLAW approved. Animals in all 3 studies were kept under controlled, recorded, environmental conditions of humidity, temperature, and light (12-h light cycle). For all 3 studies, animals of the same sex and study group per cage were co-housed, except for brief, procedure-related periods. Animals were provided with sensory and cognitive environmental enrichment, including manipulatable objects and foraging devices. Three times a day, animals were fed a standard NHP diet consisting mainly of high-protein monkey biscuits but also including PRIMA-Treats^®^, a soft dough diet, and a selection of fresh fruit, peanuts, cereals, or other treats. Tap water was provided *ad libitum* through an automated system. Animal well-being was monitored daily by husbandry staff, and routine animal health surveillance, including evaluation of blood chemistry and hematology, was provided by veterinary staff. Pre-set humane endpoints were used by a veterinarian to define study-unrelated sacrifice criteria. All measures were taken to minimize pain, distress, and suffering, and all procedures were performed by trained personnel.

### Study design and animal procedures

For study 1 and study 3, all animal procedures were performed under anesthesia either with ketamine (10–15 mg/kg intramuscularly) or Domitor (0.015 mg/kg intramuscularly). For study 2, animals were trained; therefore, vaccination and blood sampling were performed without the use of anesthesia.

Animals were assigned to the study treatment groups based on the A-series vaccine administered, receiving a vector backbone–homologous regimen matching the initial regimen for the B-series (e.g., an Ad26/Ad26 regimen twice). The initial vaccinations were given at either 12-week (studies 1 and 2) or 8-week (study 3) intervals. Treatment groups in the present study were named according to the administered vector backbones, with “rep” indicating the sequence was identical to that received previously. The interval between the last dose of the first study (A-series) and the first dose of the subsequent vaccination (B-series) was 55 weeks for study 1, 26 weeks for study 2, and 57 weeks for study 3 (Fig. 1).

Animals in study 1 were divided into 4 study groups, with 4 to 5 animals per group (Supplementary Fig. 1). The animals received 2 doses (5 × 1010 vp) of either Ad26.ZEBOV (2-dose homologous regimen, Ad26/Ad26 rep group) or Ad26.ZEBOV followed by Ad35.ZEBOV (2-dose heterologous regimen, Ad26/Ad35 rep group), with an 8-week interval between doses. Control animals who had not received any prior treatment received either a 2-dose homologous Ad26/Ad26 regimen (Ad26/Ad26 group) or a 2-dose heterologous Ad26/Ad35 regimen (Ad26/Ad35 group).

Animals in study 2 were divided into 2 study groups (pre-exposed or unexposed), with 6 animals per group (Supplementary Fig. 1). Both groups received a 2-dose heterologous vaccination regimen of 5 × 1010 vp of Ad26.SUDV at dose 1, followed 8 weeks later by 108 vp units (IFU) of MVA-BN-Filo (Ad26/MVA rep group and Ad26/MVA group).

Animals in study 3 were divided into 2 study groups (pre-exposed or unexposed), with 6 animals per group (Supplementary Fig. 1). Both groups received a 2-dose heterologous vaccination regimen of 5 × 1010 vp of Ad26.Mos4.HIV, followed 12 weeks later by 108 vp units (IFU) of MVA-mBN414A (Ad26/MVA rep group and Ad26/MVA group).

All vaccines were administered in a 0.5-mL volume intramuscularly in the quadriceps with the indicated vector particle-doses in formulation buffer. Venous blood for PBMC isolation or serum was collected from the femoral vein. Blood volumes taken did not exceed 12 mL/kg within 30 days, and a maximum of 9 mL/kg at each individual bleeding time point.

### Processing of peripheral blood

Serum samples were prepared from clotted blood drawn into serum tubes after spinning at 1900 G for 5 min at room temperature (RT). Serum was stored at –80 °C until time of analysis. PBMCs were isolated from whole blood drawn into anticoagulant-containing tubes (ethylenediaminetetraacetic acid [EDTA]) by Ficoll density gradient centrifugation. Blood was diluted 1:1 with Dulbecco’s phosphate-buffered saline (D-PBS) without Ca2+ or Mg2 + (Quality Biological, Gaithersburg, MD, USA), underlayed with an equal volume of Ficoll-Paque Plus (GE Healthcare, Little Chalfont, UK), spun at 1750 G for 40 min at RT. Buffer layers were transferred into a fresh tube, washed 3 times with D-PBS, and spun at 393 G for 5 min at RT. When needed, lysis of residual red blood cells (RBCs) in RBC Lysis Solution (Qiagen, Hilden, Germany) or ACK lysis buffer (Lonza Bio Whittaker) for 10 to 15 min at RT was performed. Lysis was stopped by addition of excess D-PBS, and tubes spun at 1750 G for 5 min at RT. Viable cell numbers were subsequently determined by trypan blue exclusion using a Countess Automated Cell Counter (Thermo Fisher Scientific, Hampton, NH, USA) or using ViaCount reagent with a third-generation Guava^®^ Easycyte™ cytometer (Luminex, Austin, TX, USA). For cells processed directly for IFNγ ELISpot, cells were adjusted to a concentration of 2 × 106 cells/mL in RPMI-10 (RPMI complemented with 10% fetal bovine serum [FBS]; Thermo Fisher Scientific), 10 mM HEPES buffer (Quality Biological), 2 mM L-glutamine (Quality Biological), and 100 ug/mL penicillin/streptomycin (Quality Biological) and kept on ice until analysis. Alternatively, cells were adjusted to a concentration of 5 × 106 cells/mL in CryoStor^®^ (Biolife Solutions, Bothell, WA, USA), and samples were aliquoted and transferred into liquid nitrogen until use.

Frozen cells were thawed in a 37 °C water bath, washed with RPMI-10, spun at 400 G for 5 min at RT, and subsequently washed twice. Cells were counted as indicated previously using ViaCount reagent with a Guava^®^ cytometer. Cells were adjusted to a concentration of 107/mL and cultured in T-25 flasks in a 37 °C, water-jacked 5% CO_2_ incubator for 18 to 24 h. Following incubation, cells were counted using ViaCount reagent with a Guava^®^ cytometer, adjusted to a concentration of 2.5 × 106 cells/mL in RPMI-10, and kept on ice until analysis.

### Adenoviral neutralization assay

Ad26 Nab titers in serum were assessed using a luciferase-based virus neutralization assay (VNA)^50^. Briefly, heat-inactivated Cynomolgus Macaque serum samples was 2-fold serial diluted starting at a 1:32, 1:64 or 1:128 dilution for Ad26 (depending on the study, see figure legends for the exact start dilution). E1/E3 deleted Ad26-luciferase reported constructs were combined with serial diluted serum into a Tissue Culture treated Black and White Isoplate-96 (Perkin Elmer, Nederland B.V) at 500 to 1000 vp/cell. Plates were incubated for 30 minutes at RT, before A549 human lung carcinoma cells (ATCC^®^ CCL-185™, American Type Culture Collection, Manassas, VA, USA) were added at a density of 1×10^4^ cells/well. After incubation for 20 to 24 hours at 37 °C and 10% CO_2_, luciferase activity was measured using the Neo-Lite Luciferase Assay System (Perkin Elmer, Waltham, MA, USA) and a BioTek Synergy Neo luminescence counter (BioTek/Agilent, Santa Clara, CA, USA) or EnVision multimode plate reader (Perkin Elmer). A 90% neutralization titer (IC_90_) was defined as the maximum serum dilution that neutralized 90% of luciferase activity. Each serum sample was analyzed in duplicate.

### IFNγ ELISpot

Antigen-specific, IFNγ-secreting T cells were enumerated in isolated PBMCs using an ELISpot kit specific for monkey IFNγ (Monkey IFNγ ELISpotPRO; MabTech, Nacka Strand, Sweden). Plates pre-coated with an NHP IFNγ-specific capture antibody (clones GZ-4 or MT126L) were washed 4 times with sterile D-PBS (180 µL/well) and blocked with RPMI-10 (200 µL/well) for 30 min at 37 °C and 5% CO_2_. After removal of the blocking buffer, PBMCs in RPMI-10 were seeded at 2 to 5 × 105 cells/well and stimulated with peptide pools reconstituted in dimethylsulfoxide (DMSO), consisting of 15-mers overlapping by 11 amino acids at a final concentration of 2 µg/mL for 18 to 20 h at 37 °C and 5% CO_2_, in a final volume of 200 µL.

For study 1, 2 peptide pools covering the EBOV GP protein N-terminal and C-terminal sequences were used to limit the number of peptides per pool (43–58 peptides/pool). The results of the N-terminal and C-terminal pools for EBOV GP were pooled for reporting purposes.

In addition, 5 peptide pools containing shared and specific peptides for Ad26 and Ad35 hexon protein were used. Responses are reported as either Ad26-hexon specific (sum of 2 Ad26-specific peptide pools) or pan-Adeno Hexon response (sum of the responses to the 5 peptide pools).

For study 2, 2 peptide pools covering the SUDV Gulu GP protein N-terminal and C-terminal sequences were used to limit the number of peptides per pool (43–58 peptides/pool). Peptides that overlapped with more than 9 consecutive amino acids within the EBOV Mayinga, SUDV Gulu, and TAFV strains or MARV Angola and Ravn strains were combined into a consensus pool, SUDVcon (~100 peptides/pool). The responses given in the figures are a sum of the background-subtracted responses induced to the 3 peptide pools (N-terminal and C-terminal pools and SUDVcon for SUDV GP).

For study 3, individual peptide pools covering the Env (Env-1, Env-2, and Env-3), the Gag (Gag-1 and Gag-2), the Pol (Pol-A, Pol-B, and Pol-C) were used^25^. The total Env response given in the figures is a sum of the background-subtracted response induced to the 3 individual sub-Env pools (Env-1, Env-2, and Env3). Likewise, the total Gag and total Pol response is a sum of the responses elicited to the individual sub-Gag pools and sub-Pol pools after background subtraction, respectively.

RPMI-10 supplemented with 0.005% to 0.33% DMSO served as a medium control and a 1/1000 dilution of α-CD3 antibody or 5.5 mg/mL phytohemagglutinin in RPMI-10 served as a positive control. After removal of the cell suspension, wells were washed 5 times with PBS + at RT and subsequently incubated with IFNγ-detector antibody conjugated to alkaline phosphatase (clone 7-B6-1-ALP, 1:200 in PBS + 0.5% FBS) for 2 h at RT. Plates were washed 5 times with PBS + at RT, and spots developed for 15 min in the dark at RT using a 5-bromo-4-chloro-3’-indolyphosphate p-toluidine/nitro-blue tetrazolium chloride solution filtered through a 0.45-µm filter. The development was stopped by washing extensively with tap water. Plates were air dried for at least 24 h before spots were counted on an ImmunoSpot S5 ELISpot plate reader (C.T.L. Europe GmbH, Bonn, Germany) or A·EL·VIS ELISpot plate reader (A·EL·VIS, Hannover, Germany), and counting was done with Eli·Analyse ELISpot Image Analysis software (A·EL·VIS). All samples were analyzed in either duplicate or triplicate. Mean SFU/106 cells were calculated from the replicate measurements, followed by individual background subtraction of the mean medium control values from the mean peptide-stimulated values. For antigens covered by >1 peptide pool, background-subtracted mean peptide–stimulated values were summed per animal per time point. Based on historical data, the background/threshold was empirically set at 50 SFU/106 PBMC. Values below the threshold of 50 SFU/106 PBMC were set at half that threshold (25 SFU/106 PBMC) for the purpose of graphical representation. For calculation of the fold-change, values below the threshold of 50 SFU/106 PBMC were set at this threshold. To calculate the fold-change from pre–dose 1 to peak response post–dose 1, the peak response post–dose 1 per animal was divided by the response measured pre-dosing (all studies, week –2). Similarly, to determine the fold-change from pre–dose 2 to peak response post–dose 2, the peak response post–dose 2 per animal was divided by the response measured pre-dosing (study 1, week 8; study 2, week 4; study 3, week 12).

### Determination of EBOV GP–specific IgG in serum by ELISA

Total serum IgG targeting GP of EBOV was assessed by an ELISA that was qualified and validated for human sera^46^. Briefly, Maxisorp™ 96-well plates (Nunc-Immuno) were coated overnight at 4 °C with Galanthus Nivalis Lectin (GNA; Sigma-Aldrich, Burlington, MA, USA) diluted in PBS at 10 μg/mL. Remaining lectin solution was removed and 200 μL PBS/10% FBS added for 90 min at RT. The plates were washed 5 times with PBS/0.2% Tween20 (PFS-T; Sigma-Aldrich) at RT, coated with supernatant-containing recombinant filovirus GP for 90 min at RT, and then washed again. Serum from NHPs was serially diluted (starting dilution, 1:50) in sample buffer (PBS/0.2% Tween/1% FBS). 100 μL of diluted sample was transferred to the coated Maxisorp™ 96-well ELISA plates, incubated for 90 min at RT, and washed 5 times with PBS/0.2% Tween20 at RT. Bound IgG was detected with goat–anti-human IgG (H + L) conjugated to horseradish peroxidase (MilliporeSigma, Burlington, MA, USA). The substrate Sigma fast o-Phenylenediamine dihydrochloride (OPD, Sigma-Aldrich, Burlington, MA, USA) was added for 10 min at RT. The enzymatic reaction was stopped by addition of 3 M H_2_SO_4_ and measured at 492 nm. IC_50_ values were calculated by 4-parameter curve-fit and compared against a filovirus GP strain-specific reference serum and expressed as ELISA units (EU) /mL.

### Determination of SUDV GP–specific IgG in serum by ELISA

Total serum IgG targeting GP of SUDV Gulu was determined by ELISA. Maxisorp™ 96-well plates were coated overnight at 4 °C with purified SUDV GP protein (produced internally at Janssen Vaccine & Prevention) diluted in 20 mM Tris-HCl solution at a concentration of 0.25 μg/mL. After washing 3 times with 200 μL PBS-T at RT, plates were blocked with 180 μL PBS/10% FBS at RT for 90 min. The plates were washed 3 times as indicated previously with PBS-T. NHP serum was serially diluted (3-fold steps) in sample buffer starting at a dilution of 1:45 (PBS/0.2% Tween/1% FBS, sample buffer) in round-bottom polypropylene plates (Nunc Cat#267245).

100 μL of diluted sample was transferred to Maxisorp™ 96-well ELISA plate and incubated at RT for 60 min. Plates were washed 3 times with PBS-T as indicated previously. Bound IgG was detected with goat–anti-human IgG (H + L) conjugated to horseradish peroxidase (MilliporeSigma, Burlington, MA, USA), diluted 1:8000 in sample buffer and incubated for 1 h at RT. Plates were washed 3 times with 200 μL PBS-T. 100 μL of Sigma Fast OPD solution (Sigma-Aldrich, Burlington, MA, USA) was added and incubated for 10 min at RT. The reaction was stopped using H_2_SO_4_ and measured at 492 nm. Endpoint concentrations were compared against a filovirus GP strain–specific reference serum and expressed as EU/mL.

### Determination of Env Clade C and Mos1-specific IgG in serum by ELISA

Antibody binding to the Clade C gp140 and mosaic gp140 antigens was determined by ELISA^51^. Briefly, antigen (HIV_Env_C_C97ZA and HIV_Env_Mos1^24^) was coated at 1 μg/mL in PBS and serum samples were tested undiluted, resulting in 1/10 serum dilution in the final ELISA plate, and incubated on plates. Binding antibody was determined using horseradish peroxidase-conjugated detection antibody mouse–anti-human IgG (Jackson Cat#209-035-011, 1:20,000) and SureBlue TMB (SeraCare 5120-0047; Kirkegaard & Perry Laboratories). The final concentration of each sample was calculated using Gen5 software (BioTek/Agilent). The concentration is equivalent to the back-calculated concentration of the measured OD450 value onto the 4PL curve-fit of the standard curve^24^.

### EBOV pseudovirus neutralization assays

The filovirus pseudovirion VNA was performed as follows^25^, and pseudovirus preparations were generated by co-transfection of human embryonic kidney (HEK) 293 cell cultures, with a replication-defective retroviral vector containing a luciferase gene along with an expression vector containing EBOV Makona GP sequence. Pseudovirus stocks were generated and characterized for suitability to assess EBOV-specific neutralization. Pseudoviruses were incubated with serial dilutions of serum samples and used to infect HEK293 cell cultures. Each serum sample was serially diluted 10 times (4-fold), starting from a dilution of 1:40. The ability of serum to neutralize EBOV pseudovirus infectivity was assessed by measuring luciferase activity ~72 h post–viral inoculation versus a control infection using a murine leukemia virus envelope–pseudotyped virus. IC_50_ values were expressed as the reciprocal of the serum dilution that inhibited the virus infection by 50%.

### RSV A2 Virus neutralization assay

VNA against RSV A2 was determined on serum of animals from study 2 using recombinant luciferase expressing RSV viruses^9^. Five thousand A549 human lung carcinoma cells were added to each well of 96-well white half-area plates (Greiner Bio-One, Frickenhausen, Germany) containing 2.5 × 10^4^ SFU/well of RSV-A2 viral particles encoding a luciferase reporter gene (resulting in a multiplicity of infection of 5), together with serial dilutions of individual heat-inactivated cynomolgus macaque serum. After incubation for 20 h at 37 °C and 10% CO_2_, luciferase activity in lysed cells was measured using the Neo-Lite Luciferase Assay System (Perkin Elmer) on a BioTek Synergy Neo luminescence counter (BioTek/Agilent). IC_50_ values were defined as the maximum serum dilution that neutralized luciferase activity by 50%. Each serum sample was analyzed in duplicate.

### Statistical analyses

Immunologic parameters (i.e., ELISA, VNA, ELISpot) were log-transformed. Two types of analysis were performed: (1) between group comparisons per time point per study using ANOVA for potentially censored values (Tobit model) and (2) within group comparisons of fold-changes over time using ANOVA. The fold-changes in response after the A-series and B-series vaccination (i.e., pre–dose 1 vs post–dose 1 and pre–dose 2 vs post–dose 2) for all immunologic parameters (i.e., ELISA, VNA, ELISpot) were calculated per animal and then log-transformed. For study 3, in addition to the comparisons per antigen, the average fold-changes over the Mos1 and Clade C antigens (ELISA) and over the Gag, Pol, Env antigens (ELISpot) were calculated and analyzed. Vaccine regimens were subsequently compared using ANOVA, both per study and pooled across studies. *P* < 0.05 were considered statistically significant, and a Bonferroni correction for 2 comparisons was applied for the analysis of study 1.

### Reporting summary

Further information on research design is available in the Nature Research Reporting Summary linked to this article.

## Supplementary information

Supplemental Appendix

REPORTING SUMMARY

## Data Availability

The data that support the findings of this study are available from the corresponding author upon reasonable request.

## Code availability

No custom code or mathematical algorithm was used for the analysis of the data.
