# Supplementary material for: Sequential use of Ad26-based vaccine regimens in NHP to induce immunity against different disease targets
Source: NPJ Vaccines. 2022 Nov 15;7:146. doi: 10.1038/s41541-022-00567-w (PMC9664441; doi:10.1038/s41541-022-00567-w)
Supplement: Supplementary file 1 — Supplemental Appendix [file 41541_2022_567_MOESM1_ESM.pdf]

SUPPLEMENTARY INFORMATION

Sequential use of Ad26-based vaccine regimens in NHP to induce immunity against different disease targets

Selina Khan, Nadine C. Salisch, Ana Izquierdo Gil, Satish Boedhoe, Karin Feddes-de Boer, Jan Serroyen, Hanneke Schuitemaker, Roland C. Zahn

a

Study 1	A-series dosing		Weeks between A- and B-series dosing	B-series dosing	
Group	Dose 1 (dose, vp)	Dose 2 (dose, vp)		Dose 1 (dose, vp)	Dose 2 (dose, vp)
26/26 rep (n=5)	Ad26.RSV.FA2 ($5e^{10}$)	Ad26.RSV.FA2 ($5e^{10}$)	55	Ad26.ZEBOV ($5e^{10}$)	Ad26.ZEBOV ($5e^{10}$)
26/35 rep (n=5)	Ad26.RSV.FA2 ($5e^{10}$)	Ad35.RSV.FA2 ($5e^{10}$)	55	Ad26.ZEBOV ($5e^{10}$)	Ad35.ZEBOV ($5e^{10}$)
26/26 (n=4)	NA	NA	NA	Ad26.ZEBOV ($5e^{10}$)	Ad26.ZEBOV ($5e^{10}$)
26/35 (n=5)	NA	NA	NA	Ad26.ZEBOV ($5e^{10}$)	Ad35.ZEBOV ($5e^{10}$)

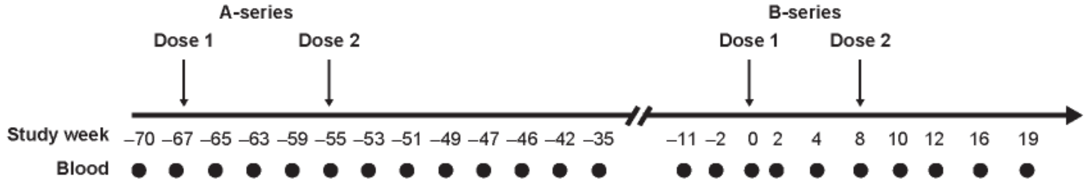

b

Study 2	A-series dosing			Weeks between A- and B-series dosing	B-series dosing	
Group	Dose 1 (dose, vp)	Dose 2 (dose, vp)	Dose 3 (dose, ifu)		Dose 1 (dose, vp)	Dose 2 (dose, ifu)
26/26/MVA rep (n=6)	Ad26.RSV.FA2 ($5e^{10}$)	Ad26.RSV.FA2 ($5e^{10}$)	MVA.RSV.FA2 ($1e^{8}$)	26	Ad26.SUDV ($5e^{10}$)	MVA-BN-Filo ($1e^{8}$)
26/26/MVA (n=6)	NA	NA	NA	NA	Ad26.SUDV ($5e^{10}$)	MVA-BN-Filo ($1e^{8}$)

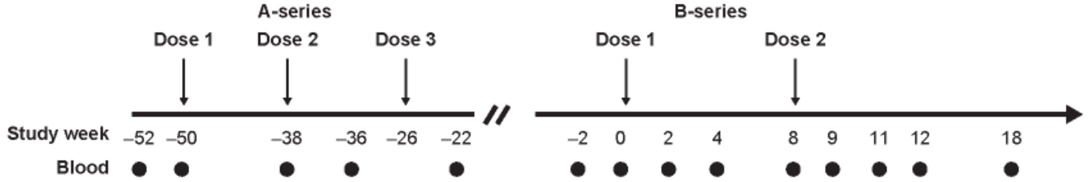

c

Study 3	A-series dosing		Weeks between A- and B-series dosing	B-series dosing	
Group	Dose 1 (dose, ifu)	Dose 2 (dose, vp)		Dose 1 (dose, vp)	Dose 2 (dose, ifu)
Ad26/MVA rep (n=6)	MVA-BN-Filo ($1e^{8}$)	Ad26.ZEBOV ($5e^{10}$)	57	Ad26.Mos4.HIV ($5e^{10}$)	MVA.mBN414A ($1e^{8}$)
Ad26/MVA (n=6)	NA	NA	NA	Ad26.Mos4.HIV ($5e^{10}$)	MVA.mBN414A ($1e^{8}$)

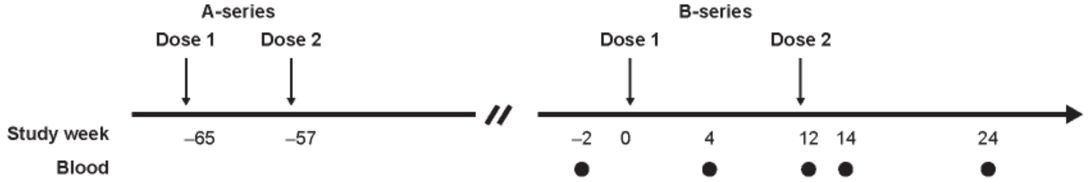

Supplementary Figure 1. Immunization regimens, timelines, and sampling time points per study. Filled circles represent a sampling time point. **(a)** Study 1: During the A-series, animals received homologous Ad26/Ad26 or heterologous Ad26/Ad35 viral vectors encoding RSV.FA2 (groups referred to as “26/26 repeat [rep]” and “26/35 rep,” respectively) or were untreated (groups referred to as “26/26” and “26/35,” respectively). Fifty-five weeks later, animals received homologous Ad26/Ad26 or heterologous Ad26/Ad35 viral vectors encoding the EBOV GP antigen in the B-series. **(b)** Study 2: During the A-series, animals received heterologous Ad26/MVA viral vectors encoding RSV.FA2 (group referred to as “26/MVArep”) or were untreated (group referred to as “26/MVA”). Twenty-six weeks later, animals received heterologous Ad26 viral vector encoding the SUDV (Sudan Gulu) GP antigen and MVA encoding SUDV, EBOV GP, or MARV GP (MVA-BN-Filo) (B-series). **(c)** Study 3: During the A-series, animals received heterologous MVA-BN-Filo vector and Ad26 vector encoding EBOV GP (group referred to as “26/MVArep”) or were untreated (group referred to as “26/MVA”). Fifty-seven weeks later, animals received heterologous Ad26.Mos4.HIV and MVA.mBN414A viral vectors encoding HIV Env, Gag, and Pol antigens (B-series). The dose of Ad26- and Ad35-vaccine vectors was 5×10^{10} vp, and the dose of MVA was 10^{8} IFU. All vaccines were administered by intramuscular injection. PBMCs and serum were collected over the course of the study for immunologic assays.

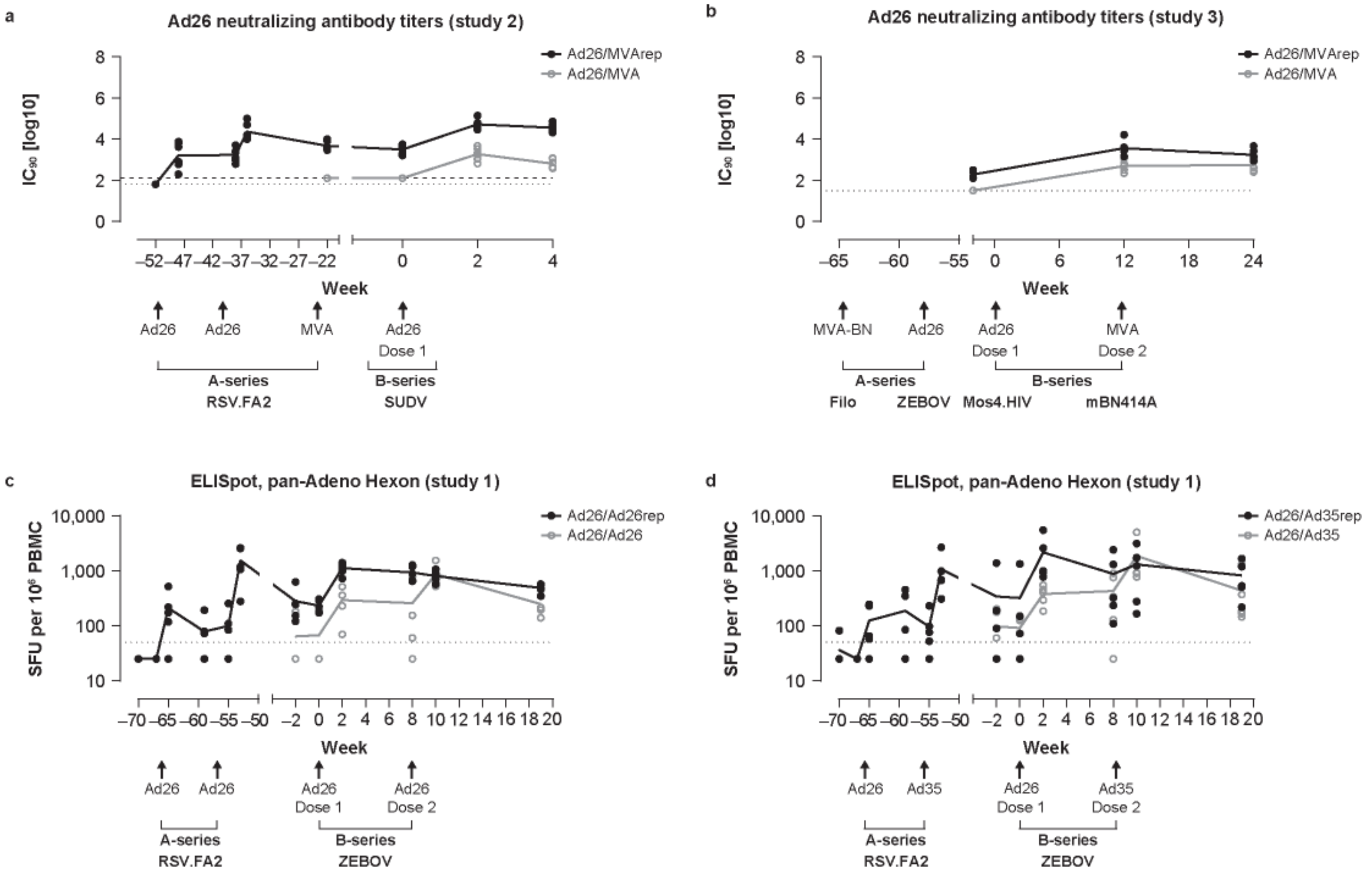

Supplementary Figure 2. Ad26 Neutralizing antibodies and pan-Adeno hexon-specific T-cell responses in cynomolgus macaques receiving multiple doses of Ad26 viral vectors.

(**a,b**) Ad26 Neutralizing antibodies (Nab) responses from animals in studies 2 and 3. Each symbol represents 1 animal. (**a**) Study 2: The 2 dotted lines depict the LLoD. The upper line corresponds to a start dilution of 125 (2.1log10) for serum samples at week −22 and week 0, and the lower line corresponds to a start dilution of 64 (1.81log10) for the remaining serum samples. (**b**) Study 3: Results depicted are from baseline (week −2) prior to dosing with Ad26.Mos4.HIV, and post-dosing with Ad26- and MVA-encoding multiple HIV antigens at weeks 12 and 14. The dotted line depicts the LLoD and corresponds to a serum start dilution of 32 (1.51log10). The lines represent the group mean. (**c,d**) pan-Adeno Hexon-specific T-cell responses, as measured by IFNγ ELISpot using PBMCs stimulated with peptide pools covering Ad26 and Ad35 hexon sequences (study 1). The dotted line corresponds to a threshold of 50 SFU/10^6 PBMCs. The lines represent the mean group response. Animals that had received an Ad26 and/or Ad35 and/or MVA vaccine during the A-series are annotated as Ad26/Ad26rep, Ad26/Ad35rep, or Ad26/MVArep, respectively.

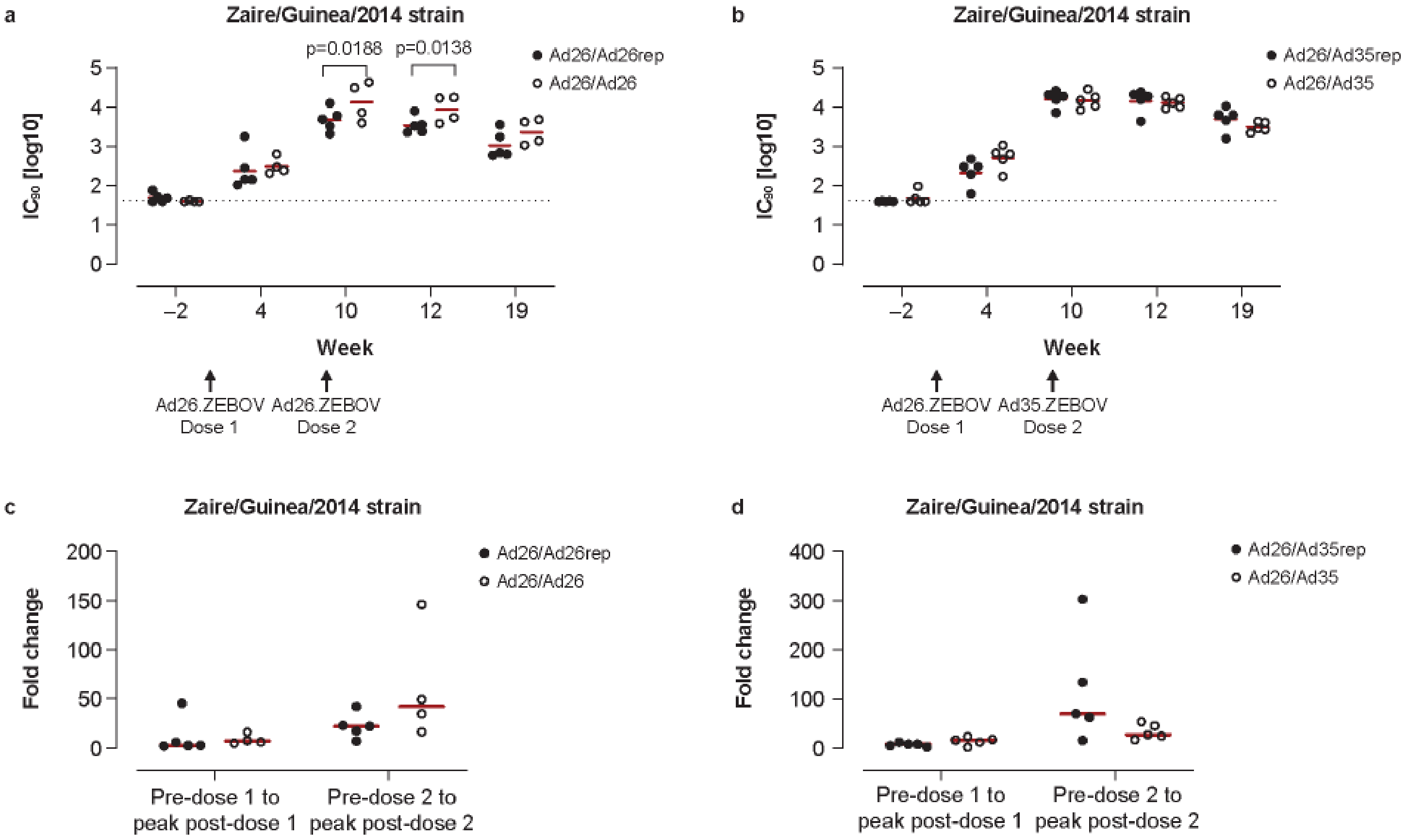

Supplementary Figure 3. Ebola Zaire/Guinea 2014 strain virus neutralization titers post–Ad26/Ad26 or Ad26/Ad35 dosing in animals with pre-existing Ad26- or Ad35-vaccine–elicited immunity.

Filovirus Nab responses against the Ebola Zaire/Guinea 2014 strain were measured using a pseudovirion VNA, as described in the Material and Methods. Shown are the antibody titers over time induced by Ad26/Ad26 (**a**) or Ad26/Ad35 (**b**) encoding EBOV GP in animals previously dosed with Ad26/Ad26 (**a**) or Ad26/Ad35 (**b**) encoding RSV.FA2 (Ad26/Ad26rep or Ad26/Ad35rep) or in unexposed animals (Ad26/Ad26 or Ad26/Ad35). The fold-change in Nab titers in animals dosed with Ad26/Ad26 (**c**) or Ad26/Ad35 (**d**) was calculated. Shown are the changes in response comparing pre–dose 1 to peak response post–dose 1 per animal and pre–dose 2 to peak post–dose 2. The red horizonal line is the geometric mean. Pairwise comparison of the difference between pre-exposed animals and unexposed animals per time point was performed for data in (**a,b**), summarized in **Supplementary Table 2**. An ANOVA was performed over the fold-changes for the data shown in (**c,d**), summarized in **Supplementary Table 3**.

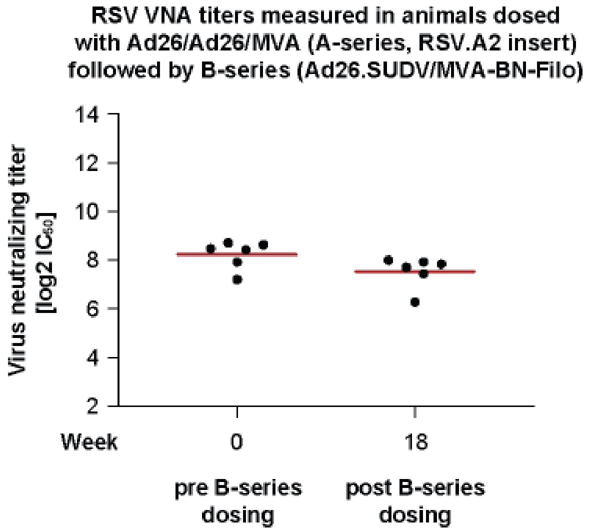

Supplementary Figure 4. RSV A2 neutralizing capacity measured in animals dosed with Ad26/Ad26/MVA (A-Series, RSV.A2 insert) followed by B-series (Ad26.SUDV/MVA-BN-Filo). Neutralizing antibody titers were determined using an RSV-A2 based VNA, as described in the Material and Methods. Data from pre-exposed animals (n=6) from study 2 are shown with each symbol represents 1 animal. Serum results depicted are from baseline (week 0) prior to dosing with Ad26.SUDV, and post-dosing with MVA-BN-Filo at week 18.

Supplementary Table 1. Statistical Results for the Magnitude of Cellular Immune Responses, Pairwise Group Comparisons

Study	Assay	Antigen	Week	Comparing treatment	With treatment	p-value	Significant
Study 1	ELISpot	EBOV	2	Ad26/Ad26rep	Ad26/Ad26	0.193	No
Study 1	ELISpot	EBOV	2	Ad26/Ad35rep	Ad26/Ad35	0.5528	No
Study 1	ELISpot	EBOV	4	Ad26/Ad26rep	Ad26/Ad26	0.6514	No
Study 1	ELISpot	EBOV	4	Ad26/Ad35rep	Ad26/Ad35	0.0046	Yes
Study 1	ELISpot	EBOV	8	Ad26/Ad26rep	Ad26/Ad26	0.5765	No
Study 1	ELISpot	EBOV	8	Ad26/Ad35rep	Ad26/Ad35	0.207	No
Study 1	ELISpot	EBOV	10	Ad26/Ad26rep	Ad26/Ad26	0.196	No
Study 1	ELISpot	EBOV	10	Ad26/Ad35rep	Ad26/Ad35	0.0016	Yes
Study 1	ELISpot	EBOV	12	Ad26/Ad26rep	Ad26/Ad26	0.066	No
Study 1	ELISpot	EBOV	12	Ad26/Ad35rep	Ad26/Ad35	0.0011	Yes
Study 1	ELISpot	EBOV	19	Ad26/Ad26rep	Ad26/Ad26	0.679	No
Study 1	ELISpot	EBOV	19	Ad26/Ad35rep	Ad26/Ad35	0.1142	No
Study 2	ELISpot	SUDV	2	Ad26/MVArep	Ad26/MVA	0.0637	No
Study 2	ELISpot	SUDV	4	Ad26/MVArep	Ad26/MVA	0.1736	No
Study 2	ELISpot	SUDV	9	Ad26/MVArep	Ad26/MVA	0.349	No
Study 2	ELISpot	SUDV	11	Ad26/MVArep	Ad26/MVA	0.3377	No
Study 2	ELISpot	SUDV	18	Ad26/MVArep	Ad26/MVA	0.211	No
Study 3	ELISpot	Env	12	Ad26/MVArep	Ad26/MVA	0.2925	No
Study 3	ELISpot	Env	14	Ad26/MVArep	Ad26/MVA	0.0974	No
Study 3	ELISpot	Gag	12	Ad26/MVArep	Ad26/MVA	0.2428	No
Study 3	ELISpot	Gag	14	Ad26/MVArep	Ad26/MVA	0.0839	No
Study 3	ELISpot	Pol	12	Ad26/MVArep	Ad26/MVA	0.7542	No
Study 3	ELISpot	Pol	14	Ad26/MVArep	Ad26/MVA	0.0494	Yes

ELISpot counts were log-transformed, and groups were compared using a Tobit model to account for possible censoring. p-values <0.05 were considered statistically significant.

Supplementary Table 2. Statistical Results for the Magnitude of Humoral Immune Responses, Pairwise Group Comparisons

Study	Assay	Antigen	Week	Comparing treatment	With treatment	p-value	Significant
Study 1	ELISA	EBOV	4	Ad26/Ad26rep	Ad26/Ad26	0.2055	No
Study 1	ELISA	EBOV	4	Ad26/Ad35rep	Ad26/Ad35	0.6917	No
Study 1	ELISA	EBOV	10	Ad26/Ad26rep	Ad26/Ad26	0.124	No
Study 1	ELISA	EBOV	10	Ad26/Ad35rep	Ad26/Ad35	0.5222	No
Study 1	ELISA	EBOV	12	Ad26/Ad26rep	Ad26/Ad26	0.5569	No
Study 1	ELISA	EBOV	12	Ad26/Ad35rep	Ad26/Ad35	0.7573	No
Study 1	ELISA	EBOV	19	Ad26/Ad26rep	Ad26/Ad26	0.3453	No
Study 1	ELISA	EBOV	19	Ad26/Ad35rep	Ad26/Ad35	0.6128	No
Study 1	PsV	EBOV	4	Ad26/Ad26rep	Ad26/Ad26	0.6808	No
Study 1	PsV	EBOV	4	Ad26/Ad35rep	Ad26/Ad35	0.0795	No
Study 1	PsV	EBOV	10	Ad26/Ad26rep	Ad26/Ad26	0.0188	Yes
Study 1	PsV	EBOV	10	Ad26/Ad35rep	Ad26/Ad35	0.8009	No
Study 1	PsV	EBOV	12	Ad26/Ad26rep	Ad26/Ad26	0.0138	Yes
Study 1	PsV	EBOV	12	Ad26/Ad35rep	Ad26/Ad35	0.7162	No
Study 1	PsV	EBOV	19	Ad26/Ad26rep	Ad26/Ad26	0.0665	No
Study 1	PsV	EBOV	19	Ad26/Ad35rep	Ad26/Ad35	0.2197	No
Study 2	ELISA	SUDV	2	Ad26/MVArep	Ad26/MVA	0.0977	No
Study 2	ELISA	SUDV	4	Ad26/MVArep	Ad26/MVA	0.0022	Yes
Study 2	ELISA	SUDV	11	Ad26/MVArep	Ad26/MVA	0.0589	No
Study 2	ELISA	SUDV	12	Ad26/MVArep	Ad26/MVA	0.46	No
Study 2	ELISA	SUDV	18	Ad26/MVArep	Ad26/MVA	0.0981	No
Study 3	ELISA	Clade C	4	Ad26/MVArep	Ad26/MVA	0.0443	Yes
Study 3	ELISA	Clade C	12	Ad26/MVArep	Ad26/MVA	0.3629	No
Study 3	ELISA	Clade C	14	Ad26/MVArep	Ad26/MVA	0.0654	No
Study 3	ELISA	Clade C	24	Ad26/MVArep	Ad26/MVA	0.1005	No
Study 3	ELISA	Mos1	4	Ad26/MVArep	Ad26/MVA	0.0323	Yes
Study 3	ELISA	Mos1	12	Ad26/MVArep	Ad26/MVA	0.3895	No
Study 3	ELISA	Mos1	14	Ad26/MVArep	Ad26/MVA	0.4965	No
Study 3	ELISA	Mos1	24	Ad26/MVArep	Ad26/MVA	0.1289	No

ELISA and PsV titers were log-transformed, and groups were compared using a Tobit model to account for possible censoring. p-values <0.05 were considered statistically significant.

Supplementary Table 3. Comparison of the Fold-changes in Immune Responses for Cellular and Humoral Responses per Study and Across Studies

Study	Assay	Antigen	Response (Log10 fold change)	Comparing treatment	With treatment	p-value	Significant
Study 1	ELISA	EBOV	pre vs post dose 1	Ad26/Ad26rep	Ad26/Ad26	0.5371	No
Study 1	ELISA	EBOV	pre vs post dose 1	Ad26/Ad35rep	Ad26/Ad35	1.0000	No
Study 1	ELISA	EBOV	pre-dose 2 vs post dose 2	Ad26/Ad26rep	Ad26/Ad26	0.1390	No
Study 1	ELISA	EBOV	pre-dose 2 vs post dose 2	Ad26/Ad35rep	Ad26/Ad35	1.0000	No
Study 1	ELISpot	EBOV	pre vs post dose 1	Ad26/Ad26rep	Ad26/Ad26	1.0000	No
Study 1	ELISpot	EBOV	pre vs post dose 1	Ad26/Ad35rep	Ad26/Ad35	0.1197	No
Study 1	ELISpot	EBOV	pre-dose 2 vs post dose 2	Ad26/Ad26rep	Ad26/Ad26	0.2580	No
Study 1	ELISpot	EBOV	pre-dose 2 vs post dose 2	Ad26/Ad35rep	Ad26/Ad35	0.0600	No
Study 1	PsV	EBOV	pre vs post dose 1	Ad26/Ad26rep	Ad26/Ad26	1.0000	No
Study 1	PsV	EBOV	pre vs post dose 1	Ad26/Ad35rep	Ad26/Ad35	0.6035	No
Study 1	PsV	EBOV	pre-dose 2 vs post dose 2	Ad26/Ad26rep	Ad26/Ad26	0.2780	No
Study 1	PsV	EBOV	pre-dose 2 vs post dose 2	Ad26/Ad35rep	Ad26/Ad35	0.1876	No
Study 2	ELISA	SUDV	pre vs post dose 1	Ad26/MVArep	Ad26/MVA	0.0189	Yes
Study 2	ELISA	SUDV	pre-dose 2 vs post dose 2	Ad26/MVArep	Ad26/MVA	0.0044	Yes
Study 2	ELISpot	SUDV	pre vs post dose 1	Ad26/MVArep	Ad26/MVA	0.3433	No
Study 2	ELISpot	SUDV	pre-dose 2 vs post dose 2	Ad26/MVArep	Ad26/MVA	0.2793	No
Study 3	ELISA	Clade C	pre vs post dose 1	Ad26/MVArep	Ad26/MVA	0.3937	No
Study 3	ELISA	Clade C	pre-dose 2 vs post dose 2	Ad26/MVArep	Ad26/MVA	0.1662	No
Study 3	ELISA	Mos1	pre vs post dose 1	Ad26/MVArep	Ad26/MVA	0.2587	No
Study 3	ELISA	Mos1	pre-dose 2 vs post dose 2	Ad26/MVArep	Ad26/MVA	0.2654	No
Study 3	ELISpot	Env	pre vs post dose 1	Ad26/MVArep	Ad26/MVA	0.8870	No
Study 3	ELISpot	Env	pre-dose 2 vs post dose 2	Ad26/MVArep	Ad26/MVA	0.0107	Yes
Study 3	ELISpot	Gag	pre vs post dose 1	Ad26/MVArep	Ad26/MVA	0.1307	No
Study 3	ELISpot	Gag	pre-dose 2 vs post dose 2	Ad26/MVArep	Ad26/MVA	0.7088	No
Study 3	ELISpot	Pol	pre vs post dose 1	Ad26/MVArep	Ad26/MVA	0.7689	No
Study 3	ELISpot	Pol	pre-dose 2 vs post dose 2	Ad26/MVArep	Ad26/MVA	0.0788	No
Study 3	ELISA	Clade C, Mos 1	pre vs post dose 1	Ad26/MVArep	Ad26/MVA	0.2891	No
Study 3	ELISA	Clade C, Mos 1	pre-dose 2 vs post dose 2	Ad26/MVArep	Ad26/MVA	0.1862	No
Study 3	ELISpot	Env, Gag, Pol	pre vs post dose 1	Ad26/MVArep	Ad26/MVA	0.6740	No
Study 3	ELISpot	Env, Gag, Pol	pre-dose 2 vs post dose 2	Ad26/MVArep	Ad26/MVA	0.0877	No

Across studies	ELISA	All antigens	Log10 fold change (pre vs post dose 1)	Ad26-Xrep	Ad26-X	0.5818	No
Across studies	ELISA	All antigens	Log10 fold change (pre-dose 2 vs post dose 2)	Ad26-Xrep	Ad26-X	0.5046	No
Across studies	ELISpot	All antigens	Log10 fold change (pre vs post dose 1)	Ad26-Xrep	Ad26-X	0.2496	No
Across studies	ELISpot	All antigens	Log10 fold change (pre-dose 2 vs post dose 2)	Ad26-Xrep	Ad26-X	0.4530	No

Fold changes in immunological response before and after each vaccination were calculated per animal and then log-transformed. For study 3, in addition to the comparisons per antigen, the average fold changes over the Mos1 and Clade C antigens (ELISA), and over the Gag, Pol, Env antigens (ELISpot) were calculated and analyzed. Vaccine regimens were subsequently compared using ANOVA. p-values <0.05 were considered statistically significant and a Bonferroni correction for 2 comparisons was applied for the analysis of study 1.
