# Supplementary material for: Sequential use of Ad26-based vaccine regimens in NHP to induce immunity against different disease targets
Source: NPJ Vaccines. 2022 Nov 15;7:146. doi: 10.1038/s41541-022-00567-w (PMC9664441; doi:10.1038/s41541-022-00567-w)
Supplement: Supplementary file 2 — REPORTING SUMMARY [file 41541_2022_567_MOESM2_ESM.pdf]

nature portfolio

Corresponding author(s): Selina Khan , Roland Zahn

Last updated by author(s): Aug 25, 2022

Reporting Summary

Nature Portfolio wishes to improve the reproducibility of the work that we publish. This form provides structure for consistency and transparency in reporting. For further information on Nature Portfolio policies, see our Editorial Policies and the Editorial Policy Checklist.

Statistics

For all statistical analyses, confirm that the following items are present in the figure legend, table legend, main text, or Methods section.

n/a	Confirmed	
☐	☒	The exact sample size (*n*) for each experimental group/condition, given as a discrete number and unit of measurement
☐	☒	A statement on whether measurements were taken from distinct samples or whether the same sample was measured repeatedly
☐	☒	The statistical test(s) used AND whether they are one- or two-sided<br>*Only common tests should be described solely by name; describe more complex techniques in the Methods section.*
☐	☒	A description of all covariates tested
☐	☒	A description of any assumptions or corrections, such as tests of normality and adjustment for multiple comparisons
☐	☒	A full description of the statistical parameters including central tendency (e.g. means) or other basic estimates (e.g. regression coefficient) AND variation (e.g. standard deviation) or associated estimates of uncertainty (e.g. confidence intervals)
☒	☐	For null hypothesis testing, the test statistic (e.g. *F*, *t*, *r*) with confidence intervals, effect sizes, degrees of freedom and *P* value noted<br>*Give P values as exact values whenever suitable.*
☒	☐	For Bayesian analysis, information on the choice of priors and Markov chain Monte Carlo settings
☒	☐	For hierarchical and complex designs, identification of the appropriate level for tests and full reporting of outcomes
☐	☒	Estimates of effect sizes (e.g. Cohen's *d*, Pearson's *r*), indicating how they were calculated

Our web collection on statistics for biologists contains articles on many of the points above.

Software and code

Policy information about availability of computer code

Data collection	Microsoft Excell for Office 365 MSO , version 2202 (Build 14931.20604), and GraphPad Prism 9.3.1 (471) were used for data collection
Data analysis	All the analyses were performed with the software R (https://www.R-project.org) version 4.1.1, and SAS 9.4 (https://www.sas.com)

For manuscripts utilizing custom algorithms or software that are central to the research but not yet described in published literature, software must be made available to editors and reviewers. We strongly encourage code deposition in a community repository (e.g. GitHub). See the Nature Portfolio guidelines for submitting code & software for further information.

Data

Policy information about availability of data

All manuscripts must include a data availability statement. This statement should provide the following information, where applicable:

- Accession codes, unique identifiers, or web links for publicly available datasets
- A description of any restrictions on data availability
- For clinical datasets or third party data, please ensure that the statement adheres to our policy

The data sharing policy of Janssen Pharmaceutical Companies of Johnson & Johnson is available at https://www.janssen.com/clinical-trials/transparency. As noted on this site, requests for access to the study data can be done by contacting the corresponding author.

Human research participants

Policy information about studies involving human research participants and Sex and Gender in Research.

Reporting on sex and gender	*Use the terms sex (biological attribute) and gender (shaped by social and cultural circumstances) carefully in order to avoid confusing both terms. Indicate if findings apply to only one sex or gender; describe whether sex and gender were considered in study design whether sex and/or gender was determined based on self-reporting or assigned and methods used. Provide in the source data disaggregated sex and gender data where this information has been collected, and consent has been obtained for sharing of individual-level data; provide overall numbers in this Reporting Summary. Please state if this information has not been collected. Report sex- and gender-based analyses where performed, justify reasons for lack of sex- and gender-based analysis.*
Population characteristics	*Describe the covariate-relevant population characteristics of the human research participants (e.g. age, genotypic information, past and current diagnosis and treatment categories). If you filled out the behavioural & social sciences study design questions and have nothing to add here, write "See above."*
Recruitment	*Describe how participants were recruited. Outline any potential self-selection bias or other biases that may be present and how these are likely to impact results.*
Ethics oversight	*Identify the organization(s) that approved the study protocol.*

Note that full information on the approval of the study protocol must also be provided in the manuscript.

Field-specific reporting

Please select the one below that is the best fit for your research. If you are not sure, read the appropriate sections before making your selection.

☒ Life sciences ☐ Behavioural & social sciences ☐ Ecological, evolutionary & environmental sciences

For a reference copy of the document with all sections, see nature.com/documents/nr-reporting-summary-flat.pdf

Life sciences study design

All studies must disclose on these points even when the disclosure is negative.

Sample size	For each of the three studies, the following applies :<br>Sample size was not determined based on a power calculation. Historical data in NHPs derived from the investigated vaccines, which could have guided statistical powering of the studies, were not available yet at the time of study design. A sample size of n=5-6 animals per group was chosen based on historical experience with Ad26-based vaccines in general, where this number provided an indication of humoral and cellular immunogenicity of vaccines in the nonhuman primate model.<br>This is considered an acceptable dataset, because it balances reasonable precision with limited use of NHP.
Data exclusions	Animals with insufficient blood volumes were excluded from certain readouts.
Replication	Samples from the study were generally analysed in individual runs, in 2-3 technical replicates. Individual runs were not repeated unless a documented technical issue in assay performance warranted re-analysis.
Randomization	NHP were assigned to groups to harmonize the groups based on sex , bodyweight and age.
Blinding	Staff at the CRO involved in animal handling, treatment, sampling and observation were not blinded since animals belonging to a treatment group were housed in dedicated rooms. Staff involved in data generation and analysis were not blinded.

Reporting for specific materials, systems and methods

We require information from authors about some types of materials, experimental systems and methods used in many studies. Here, indicate whether each material, system or method listed is relevant to your study. If you are not sure if a list item applies to your research, read the appropriate section before selecting a response.

Materials & experimental systems

n/a	Involved in the study
☐	☒ Antibodies
☐	☒ Eukaryotic cell lines
☒	☐ Palaeontology and archaeology
☐	☒ Animals and other organisms
☒	☐ Clinical data
☒	☐ Dual use research of concern

Methods

n/a	Involved in the study
☒	☐ ChIP-seq
☒	☐ Flow cytometry
☒	☐ MRI-based neuroimaging

Antibodies

Antibodies used

Study 1: goat-anti-human IgG (H+L)-conjugated to horseradish peroxidase (milipore Cat# AP504P, 1:5,000).
Study 2: goat–anti-human IgG (H+L) conjugated to horseradish peroxidase (milipore Cat# AP504P, 1:8,000).
Study 3: mouse–anti-human IgG conjugated to horseradish peroxidase (Jackson Cat#209-035-011, 1:20,000)

Validation

All used antibodies are commercially available. Validation information is available on the manufacturers' websites.

Eukaryotic cell lines

Policy information about cell lines and Sex and Gender in Research

Cell line source(s)

A549 cells originated from the ATCC (ATCC® CCL-185™), and HEK293 cells originated from the ATCC (ATCC® CCL-1573™) a master cell bank was prepared, of which working banks were prepared.

Authentication

The cell lines were obtained as authenticated. No additional authentication was performed.

Mycoplasma contamination

Mycoplasma tests are performed on the working cell banks.

Commonly misidentified lines
(See ICLAC register)

Not applicable

Animals and other research organisms

Policy information about studies involving animals; ARRIVE guidelines recommended for reporting animal research, and Sex and Gender in Research

Laboratory animals

Study 1: Five- to six-year old, healthy female cynomolgus macaques (Macaca fascicularis) of Vietnamese origin, purchased at Covance (Alice, TX).
Study 2: Four -to five -year old, healthy female cynomolgus macaques (Macaca fascicularis) of Mauritian origin, rolled over from a previous study and purchased at Charles River Edinburg.
Study 3: Four -to seven -year old, healthy male and female cynomolgus macaques (Macaca fascicularis) of Chinese origin, rolled over from a previous study and purchased at Alpha Genesis Inc.

Wild animals

N/A

Reporting on sex

Inclusion of mixed gender was only feasible for Study 3 , due to availability of male and female animals. for the study 1 and study 2 only female animals were available.

Field-collected samples

N/A

Ethics oversight

All animal research protocols were approved by the Institutional Animal Care and Use Committees at each center, and the studies were conducted in compliance with the Animal Welfare Act, Public Health Service Policy on Humane Care and Use of Laboratory Animals, and other federal statutes and regulations relating to animals and experiments involving animals.

Note that full information on the approval of the study protocol must also be provided in the manuscript.
